# Unveiling the Catalytic Mechanism and Conformational Dynamics of Guinea Pig l‑Asparaginase Type 1 for Leukemia Drug Design

**DOI:** 10.1021/acscatal.4c07791

**Published:** 2025-04-29

**Authors:** Milorad Andjelkovic, Kirill Zinovjev, Jose Javier Ruiz-Pernía, Iñaki Tuñón

**Affiliations:** † Departamento de Química Física, Universidad de Valencia, 46100 Burjassot, Spain; ‡ Instituto de Materiales Avanzados, Universidad Jaume I, 12071 Castelló, Spain

**Keywords:** asparaginases, QM/MM, binding free energy, loop motions, reaction mechanism

## Abstract

In this study, we present a computational analysis of the catalytic properties of guinea pig asparaginase type 1 (or gpASNase1), an enzyme of mammalian origin that offers a potential alternative for the treatment of acute lymphoblastic leukemia. This enzyme transforms asparagine into aspartate, depriving leukemia cells of this essential amino acid. A combination of molecular dynamics simulations, free energy calculations, and mechanistic insights based on quantum mechanics/molecular mechanics hybrid approaches was used to identify those residues contributing to the catalytic cycle of the enzyme. We dissected the contribution of enzymatic residues to substrate binding and selectivity, showing why this ASNase can degrade asparagine but not glutamine. We also studied the conformational dynamics of the enzymatic loop closing the active site, demonstrating that the substrate binding favors the closed state. The catalytic reaction mechanisms, composed of two stages, acylation and hydrolysis, were explored as well. The rate-limiting step presents a free energy barrier close to the experimental estimation and corresponds to the nucleophilic attack of enzymatic Thr19 on the carbonyl carbon atom of the substrate. Analysis of the electric field created by the protein sheds light on the role of certain residues and structural motifs in stabilizing the reaction transition state. The conclusions of this analysis are useful for rationalizing the properties of chimeras derived from gpASNase1 and predicting additional residue positions, where mutations could enhance substrate binding and loop dynamics. The results of this study enhance the understanding of gpASNase1, offering valuable insights into rational mutations and enzyme engineering for the treatment of leukemia.

## Graphical abstract

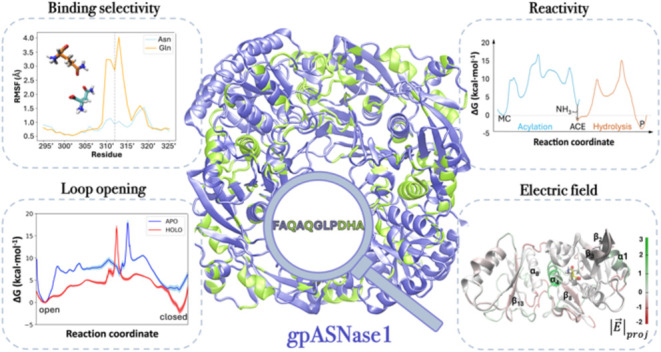

## 1 Introduction

l-Asparaginases (l-ASNases or simply ASNases) belong to a class of enzyme hydrolases that catalyze the conversion of asparagine to aspartate (see Figure 1). Since the pioneering work of Broome in the 1960s,^1^ ASNases have become a critical component in the treatment of patients suffering from acute lymphoblastic leukemia (ALL). The mechanism of action of ASNases in ALL treatment is based on the fact that acute leukemia blast cells lack the ability to synthesize asparagine by themselves and depend on the endogenous asparagine present in the blood.^2^ Therefore, the intravenous administration of an ASNase enzyme can deplete circulating asparagine in the blood, depriving blast cells of this essential nutrient and triggering their apoptosis.

**Figure 1:**
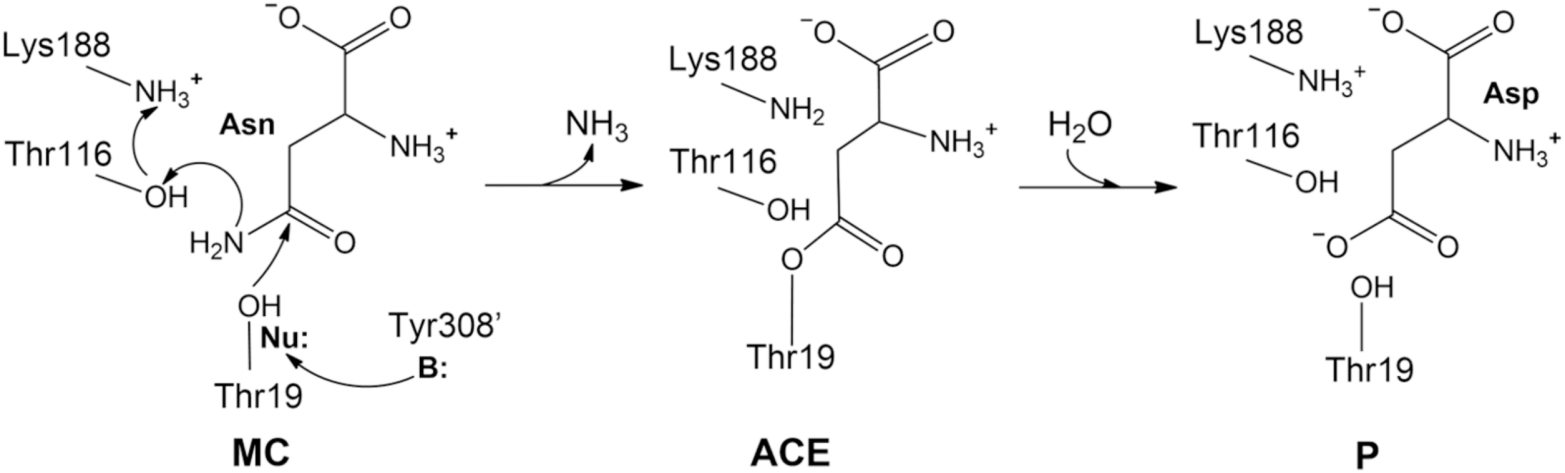
Proposed hydrolysis mechanism for gpASNase1. A nearby base (B:), Tyr308′, activates the nucleophilic residue Thr19 (Nu:) that attacks the substrate. The Lys188/Thr116 dyad protonates the leaving group substrate, forming an ammonia molecule. This leads to the covalent acyl-enzyme complex. In the second step, the product is formed (P) after the hydrolysis of this complex.

For an ASNase to be an efficient therapeutic candidate, it has to be characterized by a large binding affinity for asparagine (*K*_M_ in the low micromolar range), selectivity, and a high catalytic efficiency (large *k*_cat_).^3^ Two types of ASNases are drugs being used for ALL treatment nowadays: E. coli ASNase type 2 (EcASNase2) and Erwinia chrysanthemi ASNase type 2 (ErASNase2).^4,5^ These enzymes are structurally well-resolved and there are numerous experimental and computational analyses of their catalytic activity.^6–10^ Both enzymes exhibit very good catalytic efficiency and binding affinity toward asparagine, reasons that justify their selection. However, severe hypersensitivity reactions have been associated with their use for ALL treatment.^11,12^ Due to the bacterial origin of these enzymes, the immunogenic response can be triggered by these treatments, leading to the inactivation of the enzymes in treatments maintained over time.^13^ Naturally, the field shifted toward the possibility of replacing bacterial enzymes with mammalian ASNases. A few studies have been published to characterize human and guinea pig asparaginases type 3 (hASNase3 and gpASNase3).^14,15^ In our previous work, we have provided the theoretical basis for understanding the reaction mechanism and catalytic efficiency in hASNase3.^16^ However, regardless of their good catalytic efficiency, these enzymes suffer from quite poor binding affinity.^14,15,17^ Recently, a new type of human asparagine, hASNase1, has been identified and characterized.^18^ Although the catalytic rate of this enzyme was quite good, similar to gpASNase3 and hASNase3, it also struggled with a relatively weak binding affinity.^18,19^ Almost at the same time a new mammal ASNase, gpASNase1, has been characterized, showing properties that turn it into a potential candidate for the replacement to the bacterial enzymes used in ALL treatment.^20^

The enzyme gpASNase1 consists of two intimate dimers, coming together to form a donut-like-shaped homotetramer, spanning a total of four active sites, as shown in Figure 2. According to the X-ray structure (PDB code 5DNC, which corresponds to the Thr19Ala mutant),^21^ several residues can stabilize and orient the substrate in the active site of gpASNase1. Given that at physiological pH asparagine is found in the zwitterionic form, the negatively charged carboxylate group of the substrate is stabilized by the Ser85 backbone amino group and its side chain hydroxyl group, as well as by the backbone amino group of Asp117. Meanwhile, the positive amino group of the substrate is stabilized by the carboxylate groups of Asp84 and Asp117. The latter one is held in place by Lys188. Additionally, other residues are involved in substrate positioning at the active site. These include hydrogen bonding interactions between the side chain amino group of the substrate and the backbone carboxyl oxygen of Ala142.

**Figure 2:**
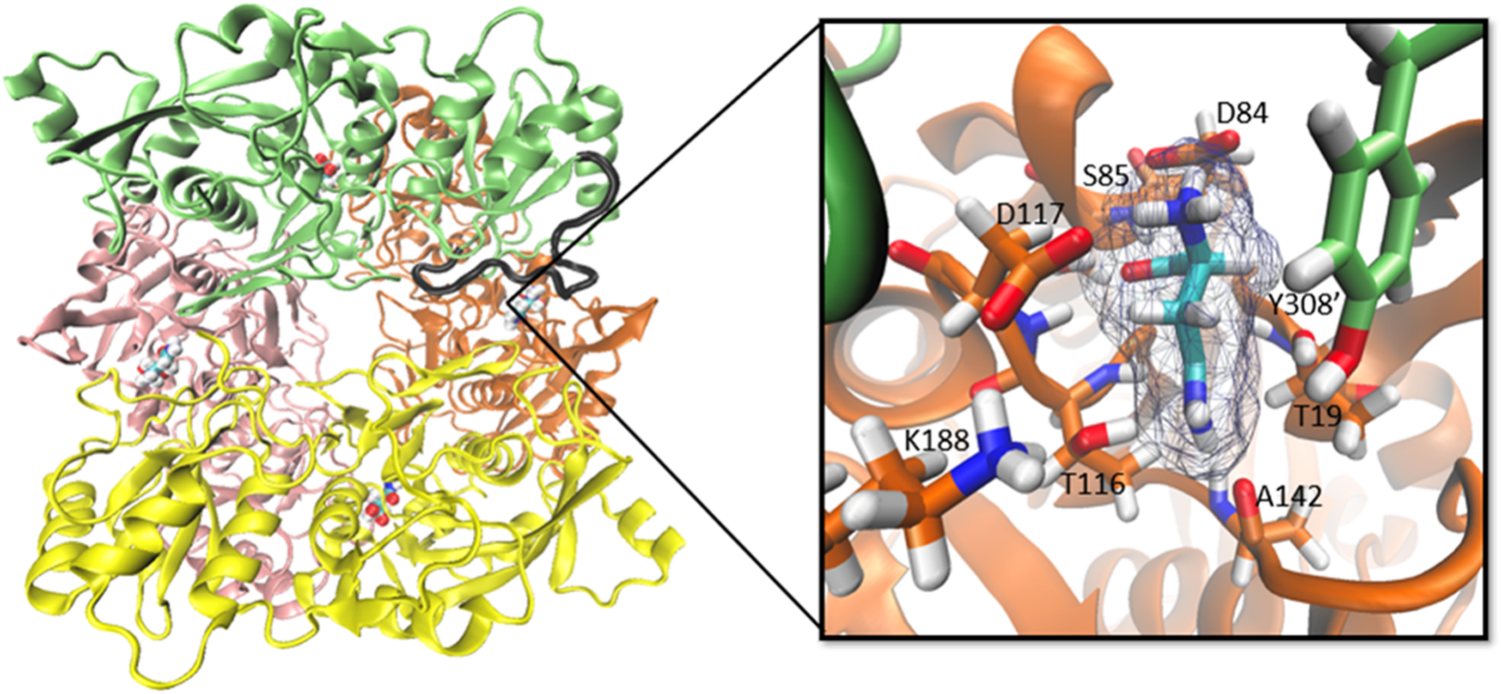
Homotetrameric structure of gpASNase1. Pink, green, orange, and yellow colors represent the four different monomers, forming a donut-like-shaped structure. Substrates in the four active sites are shown using a ball representation. One of the Tyr308′-flexible loops is represented in black color. On the right, a zoom in of one of the active sites is provided, representing the substrate and depicting some of the most important active site residues that are stabilizing the substrate in the active site. In this representation, we reverted the Thr19Ala mutation present in the X-ray structure (PDB code: 5DNC).

The proposed reaction mechanism includes two principal steps, as suggested before and schematically shown in Figure 1.^7,22^ In the first step, starting from the Michaelis complex (MC), the acyl-enzyme (ACE) complex is being formed. Thr19 gets deprotonated by Tyr308′, a residue from the adjacent protomer, and then acts as a nucleophile (Nu:) attacking the Asn substrate. This first step also involves the release of ammonia from the substrate after a proton transfer from the dyad Thr116/Lys188 to the side chain NH_2_ group of the substrate. This leads to the formation of a covalent tetrahedral acyl-enzyme intermediate (ACE). In the second step, the ACE complex gets hydrolyzed by the nucleophilic attack of a water molecule, leading to the reaction product (P), where aspartate is already formed, and active site residues recover their original protonation states.

While gpASNase1 shares a high sequence identity with hASNase1 (almost 85% when limiting to the relevant domains),^19^ this enzyme exhibits binding affinity and kinetics properties comparable to clinically effective ASNases.^19^ It has been suggested that large-scale conformational changes caused upon substrate binding and/or the allosteric cooperative behavior observed in hASNase1 might be responsible for its poor binding affinity compared to gpASNase1, in particular since neither allostericity nor large domain rearrangements have been observed in gpASNase1 and other ASNases.^19^ The most noticeable conformational change observed in gpASNase1 after substrate binding is the rearrangement of the C-terminal loop formed by residues Thr305′ and Asn316′ belonging to the other protomer of the intimate dimer (see Figure 2). Specifically, in the product-bound gpASNase1 structure, this loop is observed to act as a lid restraining the substrate into the active site.^20^ This loop contains Tyr308′, which is the residue in charge of the activation of the nucleophile during ACE formation (see Figure 1), and then we will refer to this loop as the Tyr-loop. This loop is also present in hASNase1 but containing two additional residues that could be responsible for its reduced affinity by asparagine in comparison to gpASNase1.^19^ Additionally, gpASNase1 was found to be highly selective toward asparagine in comparison to glutamine.^20^ The lack of l-glutaminase activity seems to be of a great importance for the clinical activity of ASNases.^23–25^ Due to its kinetic and binding properties and mammalian origin, there is a growing interest in the study of the gpASNase1 enzyme for its potential use in ALL treatments. A recent work addressed the reduction of the possible immunogenic response derived from its use, employing directed evolution and DNA shuffling with hASNase1 to create humanized versions of gpASNase1.^19^ Several of these humanized clones were shown to have good kinetics properties and thus are potential candidates for clinical use.^19^

Until now, very few theoretical analyses have been performed to identify the origin of the improved properties of gpASNase1 with respect to the highly similar human variant. In particular, one paper has been recently published containing a theoretical study of the reaction mechanism of gpASNase1 based in a QM/MM scheme and geometry optimizations using a dimer model for the enzyme.^7^ To our knowledge, no theoretical work has yet been done to elucidate the binding and reactivity properties of gpASNase1, considering its conformational dynamics. This work aims to elucidate the reaction mechanism, binding selectivity, and loop conformational changes of gpASNase1, identifying those residues and structural motifs that contribute to their properties. This information, combined with multiple sequence alignments, can be useful to rationalize and guide the design of ASNases to treat ALL.

## 2 Methods

### 2.1 System Preparation

The Michaelis complex structure was built starting from the PDB structure 5DNC^21^ corresponding to the N-terminal domain homotetrameric form (see Figure 2). Experimental results demonstrated that truncating the C-terminal domain of gpASNase1 does not affect its catalytic activity and K_M_ value.^19^ The starting PDB structure corresponds to the catalytically inactive mutant Thr19Ala. Therefore, to model a reactive complex, the mutation was reversed in the four protomers. The conformation of Thr19 was chosen so that the hydroxyl group is oriented toward the substrate, as observed in the 4R8L structure^20^ that corresponds to the complex of the wild-type enzyme with the product (aspartate) in the active site. Since residues 1–7 and 361–362 were not resolved in structure 5DNC, their coordinates were modeled using AlphaFold2.^26^ All water molecules and sodium ions present in the X-ray structures were preserved. Missing H atoms were added using the Protein Preparation Wizard tool of Maestro,^27^ and PROPKA3 ^28^ was used to calculate the protonation states of titratable residues at pH 7.4. Lys188 was modeled in the protonated state, as suggested from p*K*_a_ calculations although the possibility of a deprotonated form was explored using free energy methods, as described below. Similarly, a model of the gpASNase1 structure in the apo form was prepared starting from the 4R8K structure.^20^

### 2.2 Molecular Mechanics Molecular Dynamics Simulations

Molecular dynamics (MD) simulations based in a molecular mechanics (MM) force field were run using the Amber22 GPU version of pmemd.^29^ Parameters for the substrates, free Asn and Gln in their zwitterionic forms, were taken from Horn et al.,^30^ while protein amino acids were described using the ff14SB force field.^31^ In the simulations of the acyl-enzyme complex, the parameters for the covalently bound acyl-enzyme were obtained using the antechamber program in Amber22, while atomic charges were derived using the restrained electrostatic potential (RESP) method at the HF/6-31G­(d) level.^32^ The systems were solvated in a box of TIP3P^33^ water molecules using the tleap tool from AmberTools22,^29^ ensuring that protein–substrate atoms were at least 12 Å away from the simulation box boundaries. The net charge of the system was neutralized by adding sodium ions.

After system minimization and equilibration (details in the Supporting Information), three replicas of 1 μs each, initiated with different velocities, were run for both the holo and apo forms of the enzyme. Simulations were conducted in the NVT ensemble with periodic boundary conditions. Temperature was maintained at 310 K using the Langevin thermostat, and the time step was set to 2 fs using SHAKE.^34^ Electrostatic interactions were computed using particle-mesh Ewald,^35^ while a 10.0 Å cutoff radius was applied for all nonelectrostatic interactions.

To analyze the electric field effects from our molecular dynamics simulations, we used a modified version of the TUPÃ software.^36^ Given that a negative charge builds up on the carbonyl oxygen of the substrate during the enzymatic reaction (see below), the midpoint of the C=O bond was set as a probe for the calculation of the electric field and its projection, using a unit vector defined from O_δ_ to the C_γ_ of the substrate. Electric field analysis was performed on snapshots of the MD simulations of the acyl-enzyme state and the solvent cutoff radius was set to 15 Å. Additional technical details are given in the Supporting Information.

### 2.3 Molecular Mechanics Free Energy Calculations

To determine the p*K*_a_ of Lys188, thermodynamic integration (TI) was employed to estimate the free energy change along the alchemical transformation between the neutral and protonated forms, following a computational setup similar to that of He et al.^37^ The free energy was calculated by numerical integration of the derivative of the free energy with respect to the coupling parameter (λ). The free energy associated with the release of ammonia from the active site after the first chemical step of the catalytic cycle was determined using a similar procedure where the transformation is performed between ammonia in the active site and ammonia in solution. Additionally, the relative binding free energy between asparagine and glutamine has been calculated by applying an alchemical transformation between the two bound states. The thermodynamic cycles followed in all of these transformations are illustrated in Figures S1–S3. Details of this TI simulation can be found in the Supporting Information and elsewhere.^16^

In the study of the free energy profiles corresponding to the Tyr-loop conformational change from open to closed states in the apo and holo forms, thermodynamic ensembles representing the closed and open states were obtained extracting snapshots every 10 ns of the 1 μs MD simulation at each state (open and closed). These MD simulations were run using the same specifications as indicated above, but adding a light restrain (with a force constant of 10 kcal·mol^–1^·Å^–2^) to the coordinates of the substrate to keep it in the active site in the simulations of the open-holo gpASNase1. These snapshots were used as starting structures for the adaptive string method (ASM) simulations of the conformational change. ASM, developed in our research group and integrated in the Amber24 software,^38,39^ has been proven as effective in the study of conformational changes in protein loops.^40^ This approach defines several replicas of the system (the string nodes) along a hypothetical pathway connecting the two states in a space spanned by a set of collective variables (CVs). The CVs used here include backbone torsional angles to describe the conformational rearrangement of the loop backbone and distances among residues to define the displacement of the loop (see below). The positions of the nodes evolved according to the free energy gradient while being kept equidistant to ensure convergence to the minimum free energy path (MFEP). To enhance convergence, Hamiltonian replica exchange between neighbor nodes was employed. Convergence of the string is assessed using the root-mean-square deviation (RMSD) between the values of the CVs. After convergence (RMSD ∼ 0.1 amu^1/2^·Å for at least 2 ns), a path-CV (denoted as *s*) is defined to measure the position of the system along the MFEP. This path-CV is then used as the reaction coordinate for umbrella sampling (US) calculations. The free energy profile is estimated using the weighted histogram analysis method (WHAM).^41^ Force constant values employed in US calculations are determined on-the-fly to ensure a homogeneous probability density distribution of the reaction coordinate. Technical details of the ASM simulations are given in Table S5.

In order to determine the per-residue contribution to the interaction energies, we used the molecular mechanics generalized born surface area (MMGBSA) applying the MMPBSA.py script^42^ available in AmberTools22.^29^ Complex, receptor, and ligand topology files were generated using the Ante-MMGBSA.py utility,^42^ with GB solvation radii set to mbondi2 and the salt concentration set to 0.1 M. Electrostatic and van der Waals contributions to the interaction energy were calculated on a per-residue basis. Rather than using the standard MMPBSA.py decomposition scheme that distributes the interaction energy evenly between the ligand and the receptor residue, full contribution has been assigned to each residue.^43^ All MMGBSA calculations were carried out on uncorrelated and equally distanced frames (100 ps) extracted from a 1 μs trajectory of the gpASNase1 complex with the substrate. The statistical error for each residue’s contribution to the interaction energy was computed as the standard deviation of 10 average values.

### 2.4 QM/MM Simulations

Hybrid quantum mechanics/molecular mechanics (QM/MM)^44^ calculations were performed using Amber24 ^39^ coupled to Gaussian16 ^45^ for density functional theory calculations. The B3LYP functional,^46,47^ with D3 dispersion corrections^48^ and the 6-31G­(d) basis set, was employed to describe the QM subsystem. This functional was chosen based on its accuracy in previous studies of ASNases type 1 and 2.^22^ Preliminary calculations were performed at the GFN2-xTB/MM^49^ and DFTB3/MM^50^ levels. The QM subsystem included the entire substrate (Asn) and the side chains of all active site residues (Thr19, Thr116, Asp117, Lys188, and Tyr308′) involved in the reaction, placing the QM/MM boundary between C_α_ and C_β_ atoms. For the acyl-enzyme formation step, the QM subsystem included three water molecules, while in the case of acyl-enzyme hydrolysis, only two QM water molecules were needed (see below).

To explore different mechanistic proposals, the free energy profiles of the reaction mechanisms were obtained using the ASM starting from different initial guesses for each possible mechanism. In this case, the CVs used to explore all of the mechanisms were all of the distances of the bonds to be broken and formed during each stage of the mechanism (see below for the list of distances included in each reaction step). To enhance the sampling, the mass of all transferred hydrogen atoms was set to 2 amu and the time step was set to 1 fs. Other technical details for QM/MM ASM calculations are given in Table S6.

## 3 Results and Discussion

### 3.1 Analysis of Active Site Interactions with Asparagine

To study the dynamic behavior of gpASNase1, we run molecular dynamics (MD) simulations for the apo enzyme and the Michaelis complex with asparagine. A previous study of the gpASNase1 simulated one of the dimers, adding restraints to keep a catalytic conformation in the active site.^7^ In this work, the full tetramer was used in the simulations showing that, without any biases, the protein and the substrate remained stable in the four active sites in all replicas throughout the whole simulation time (1 μs), as evidenced by the time evolution of the root-mean-square deviations (RMSD) (see Figure S4) and visual inspection. In all of the simulations, the substrate was kept well-positioned in the active site, with the binding pose resembling that observed in the X-ray structures of the product and the Michaelis complex (PDB codes 4R8L and 5DNC). Relevant interactions established between the enzyme and the substrate are shown in Figure 3, while the probability distributions of the associated distances are given in Figure S5.

**Figure 3:**
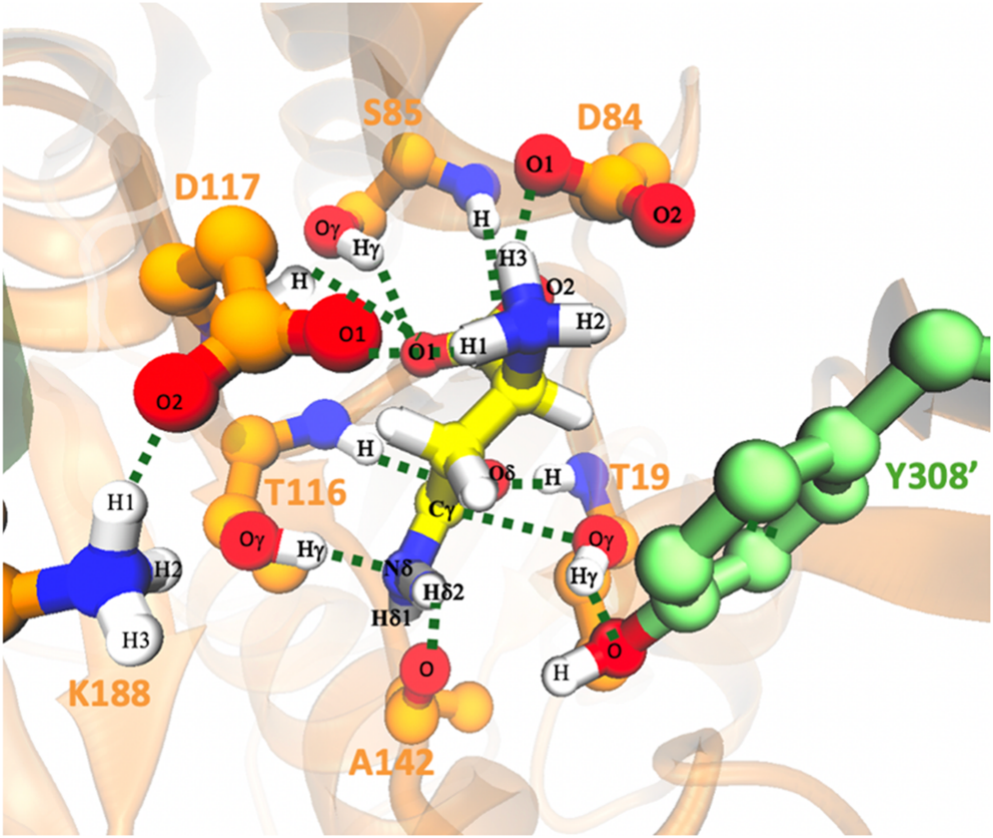
Active site of the Michaelis complex of gpASNase1 with the Asn substrate (yellow ball and sticks model) obtained from MD simulations with some important distances between the active site residues. Probability distributions of the distances shown here are given in Figure S5.

One of the controversial issues in the study of type 1 and 2 ASNases has been the protonation state of lysine present in the active site. Some of the mechanistic proposals relay on Lys188 (or the equivalent residue in other ASNases) being deprotonated, facilitating this residue to act as the base that deprotonates the nucleophile.^6,21^ Other mechanistic proposals (see Figure 1) consider that this residue is protonated and participates in the proton transfer to the amino leaving group. Our free energy calculations show that the deprotonation of this residue at the Michaelis complex requires a free energy cost of 3.7 ± 0.1 kcal·mol^–1^ at pH = 7.5 and 310 K (see the Supporting Information devoted to the calculation of Lys188 p*K*_a_). Therefore, in our simulations of the Michaelis complex, we considered Lys188 in its protonated form. As discussed below, protonated Lys188 participates in hydrogen bond interactions relevant for binding and catalysis.

According to our simulations, the stabilization of the positively charged amino group of the substrate involves strong and stable interactions with the carboxylate groups of Asp84 and Asp117 (Figures 3 and S5). Meanwhile, the negatively charged α-carboxylic group of the substrate establishes hydrogen bond interactions with the backbone amino group of Ser85 and its side chain hydroxyl group, as well as the backbone amino group of Asp117. Additionally, the carbonyl oxygen atom of the substrate forms two hydrogen bonds with backbone amino groups of Thr116 and Thr19, forming an oxyanion hole that stabilizes the negative charge on the substrate’s oxygen atom during the chemical reaction. These interactions contribute to orient the NH_2_ group of the substrate toward the bulk, facilitating the elimination of the leaving group (NH_3_) during the reaction. It is important to notice the proximity of the carbonyl carbon atom (Cγ) of the substrate to the hydroxyl oxygen of Thr19 (Oγ) (average distance of 3.46 ± 0.44 Å). As discussed below, Thr19Oγ acts as the nucleophile attacking the carbonyl carbon of the substrate to form the ACE complex.

### 3.2 Binding Selectivity

To study the preferential selectivity of gpASNase1 for Asn instead of Gln, MD simulations of the Michaelis complex with Gln were also run. Using an alchemical transformation and TI, we determined the relative binding free energy of gpASNase1 with both substrates, as described in the Supporting Information devoted to relative binding free energies and in Table S4. The relative binding free energy of gpASNase1 with Asn was found to be 6.3 ± 0.6 kcal·mol^–1^ more favorable than with Gln. This result agrees with the experimental observation of the lack of glutaminase activity in gpASNase1.^20^ In order to identify which residues are responsible for this selectivity, we employed the MMGBSA scheme to calculate the average interaction energies *E*_int_ (sum of the electrostatic and van der Waals components) of each enzymatic residue with both substrates along 1 μs simulations. The results are listed in Figure 4.

**Figure 4:**
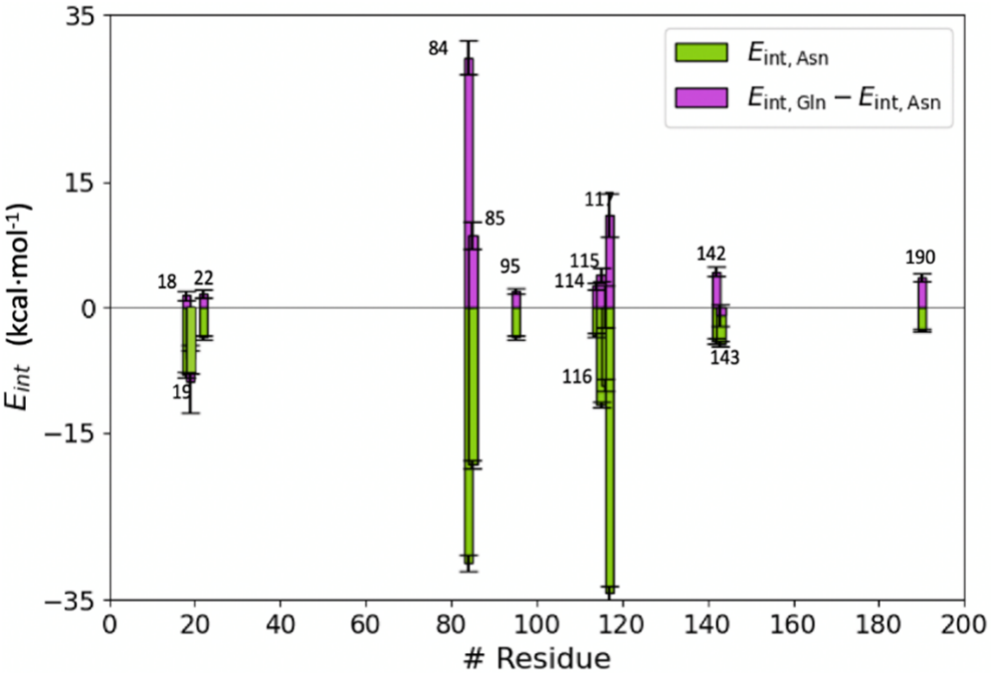
Individual average residue contributions to the interaction energy of Asn (*E*_int,Asn_ shown with green histogram bars) and differential contribution to the binding of glutamine and asparagine (*E*_int, Gln_ – *E*_int, Asn_ displayed in purple). For simplicity, only residues with |*E*_int, Asn_| values larger than 2 kcal·mol^–1^ are shown. Free energy values are expressed in kcal·mol^–1^. Error bars are calculated as the standard deviation of the mean values.

Protein residues contributing more to the binding of Asn are those with larger green histogram bars in Figure 4. These correspond to residues interacting with the charged carboxylate and amino groups of the zwitterionic substrate and include Asp84, Ser85, Thr116, and Asp117. As seen from Figure 4, Asp190 and Ala142 also contribute significantly to the binding of the substrate. The contribution of Asp190 is due to the long-range nature of the electrostatic interactions of its charged side chain. However, Ala142 has an important direct role in substrate binding: the carbonyl group of Ala142 is hydrogen bonded to the side chain amide group of the substrate, positioning its C_γ_ atom in an optimal position for the nucleophilic attack by the hydroxyl group of Thr19 (see Figure 3). Residues Thr19 and Thr116 also play an important role in the binding process. The main chain amino groups of these residues form the oxyanion hole that interacts with the carbonyl oxygen atom of the substrate.

Most of the residues responsible for Asn binding display stronger interactions with Asn than with Gln, with the largest differences being found for Asp84, Ser85, and Thr117 (see purple bars in Figure 4). To accommodate a larger substrate (Gln), the side chain of Ser84 must be oriented away from the active site. Interestingly, Thr19 interacts better with Gln than with Asn (the corresponding purple histogram bar in Figure 4 is negative). The hydroxyl groups of both Thr19 and Thr116 are oriented toward the carboxylate group of Gln, as seen in Figure 5. This shows that the resulting binding pose of Gln is not a reactive one. First, the hydroxyl group of Thr19 does not form a hydrogen bond interaction with Tyr308′, an interaction observed in the case of Asn (see Figure 5b) and this is essential for the activation of the nucleophilic attack. Second, the hydroxyl group of Thr116 is not oriented toward the NH_2_ leaving group of the substrate (see Figure 5a). This residue is a candidate to protonate the leaving amino group. Missing the Thr19–Tyr308′ interaction does not only affect the preorganization of the active site for the nucleophilic attack but also the positioning of the Tyr-loop, much more flexible when Gln is the substrate (see the root-mean-squared fluctuations of loop residues in Figure 5c).

**Figure 5:**
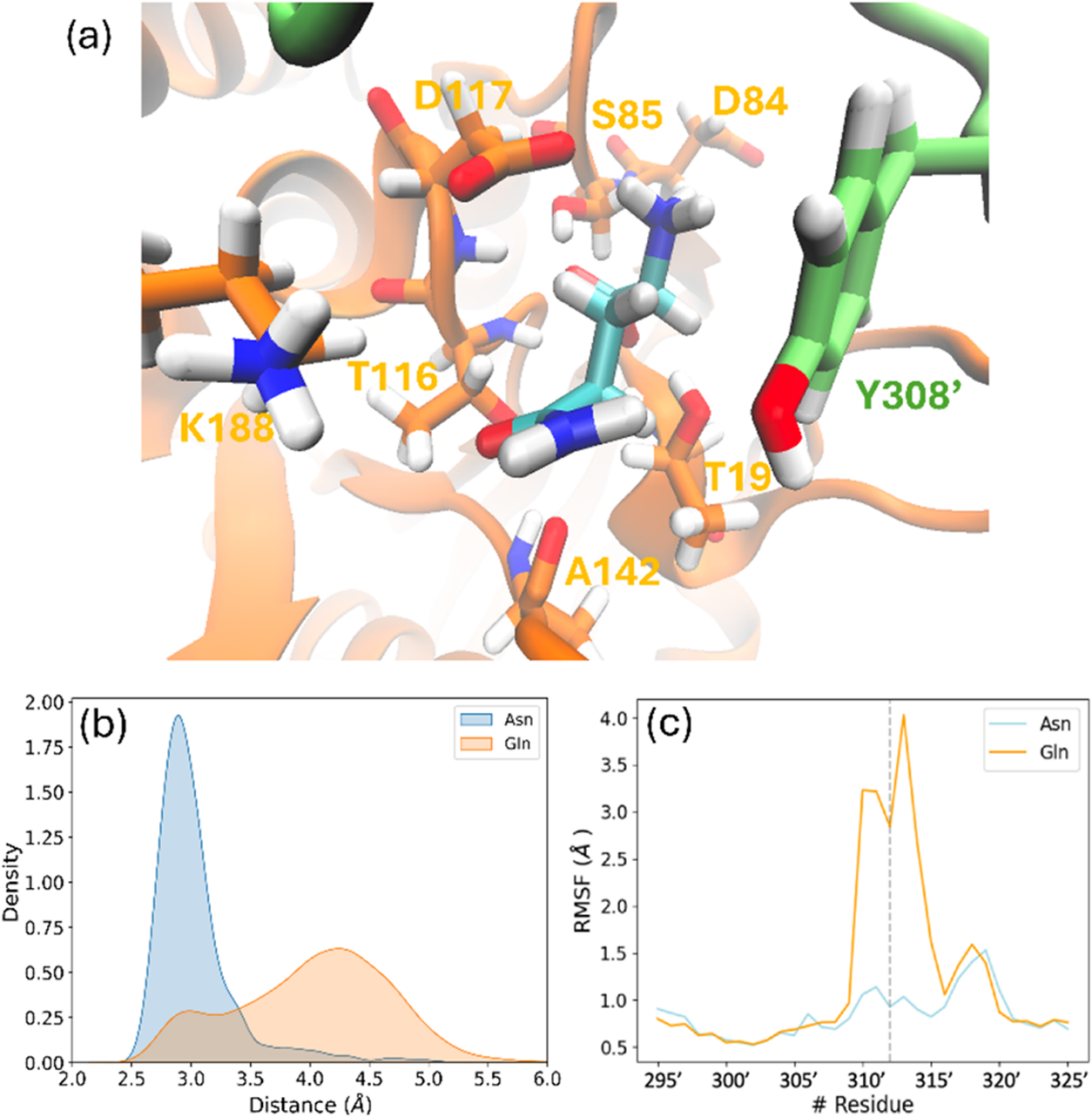
(a) Representation of the binding pose of Gln in the active site of gpASNase1 and some important residues whose contribution to the interaction energy has been found to change considerably with respect to Asn; (b) probability distribution of the Thr19–Tyr308′ distances during the 1 μs simulation in the case of Asn (blue) and Gln (orange) occupying the active site of gpASNase1; and (c) root-mean-square fluctuations of C_α_ atoms of the Tyr-loop residues in the case of Asn (blue) and Gln (orange) in the active site.

### 3.3 Conformational Change of the Tyr-loop

The Tyr-loop contains Tyr308′, a residue that is postulated to activate the nucleophilic attack of Thr19 on the substrate. This loop must be open to facilitate the binding of the substrate and then closed to reorganize the active site for the chemical step, forming the Tyr308′–Thr19 contact discussed above. To identify the coordinates that govern the conformational change of this loop, we analyzed classical MD simulations for the open and closed states in the apo and holo forms of the enzyme. Figure 6 shows the open and closed states of the loop along with five CVs (two torsional angles and three distances) selected for the simulations with the ASM. As seen from Figure 6a, in the closed state, Tyr308′ forms a hydrogen bond with the nucleophile Thr19, while in the open state the side chain of Tyr308′ is rotated to establish a hydrogen bond with the main chain carbonyl group of Pro274. These two distances, together with that associated with the intraloop hydrogen bond formed between Ala309′ and Ala313′ in the open state, were selected to drive the conformational change in our simulations.

**Figure 6:**
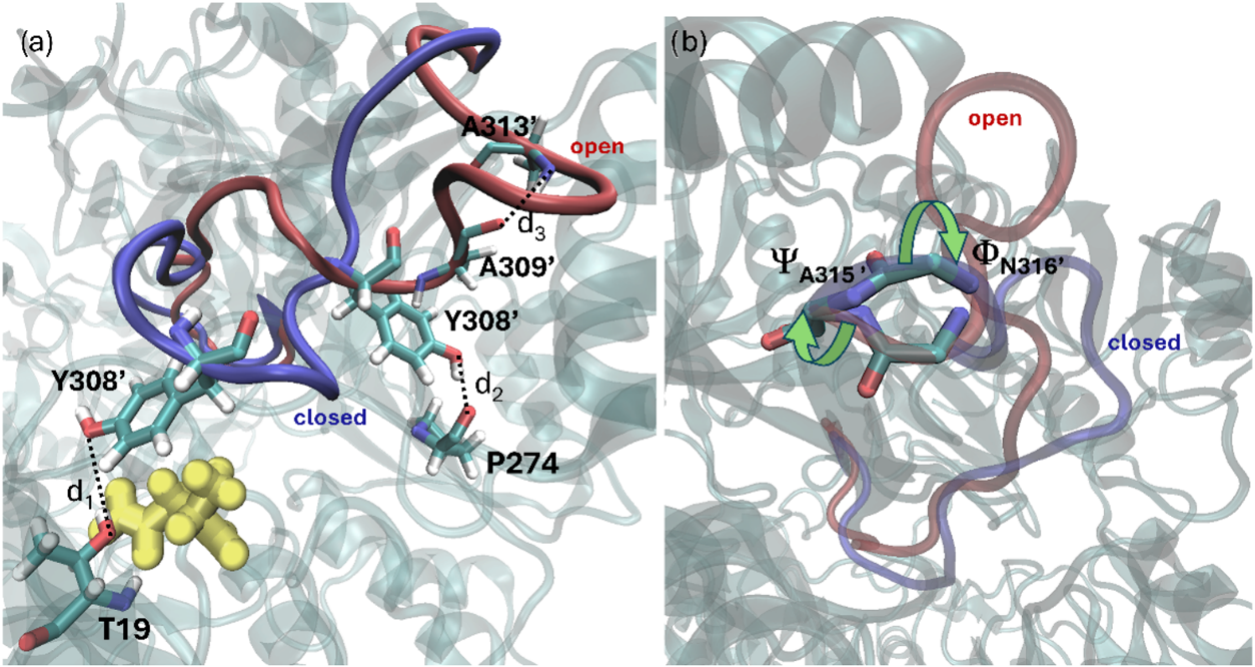
Open (red) and closed (blue) states of the Tyr-loop. (a) Residues Thr19, Pro274, Tyr308′, Ala309′, and Ala313′ are used to define the distances d_1_, d_2_, and d_3_ that differentiate the position of the loop in both states (d_1_ is the distance between Tyr308′O_γ_ and Thr19O_γ_, d_2_ the distance between Tyr308′O_γ_ and Pro274O_γ_ and d_3_ the distance between Ala309′O and Ala313′N, describing the formation of a small a-helix-like turn motif in the open state). The substrate is represented with ball and stick models in yellow color. (b) Backbone torsions around the Ala315′–Asn316′ peptide bond that define the conformational state of the loop backbone in the closed and open states.

In addition to these distances, backbone torsions also contribute to the conformational change between open and closed forms.^40^ The probability distributions for all Φ and Ψ dihedral angles of the loop backbone (residues 300–316) and for the three distances defined in Figure 6, obtained from 1 μs MD simulations of the open and closed states, are shown in Figures S6 and S7, for the apo and holo forms of gpASNase1, respectively. The only torsional angle whose distributions in the open and closed states of the loop do clearly not overlap in the holo or in the apo forms is Ψ_315′_ and, to a minor extent, the contiguous torsional angle Φ_316′_ (see Figure S8). Therefore, we chose these two torsions, which define the rotation of the Ala315′–Asn316′ peptide group, together with the aforementioned distances, as CVs to drive the simulations of the loop conformational change. A similar mechanism, with a single peptide group rotation controlling the transition between the open and closed forms, was described for the WPD-loop of the PTP1B enzyme.^40^

Using this set of five CVs, we obtained the MFEPs corresponding to the close/open conformational transition of the Tyr-loop with the ASM for both the apo and holo forms of the enzyme. The resulting free energy profiles along the path-CVs are shown in Figure 7. Figures S9 and S10 display the individual free energy profiles and the corresponding evolution of collective variables along the MFEPs for the apo and holo systems, respectively. As an additional confirmation that the set of chosen CVs was sufficient to govern the conformational change in the Tyr-loop, the open and closed structures obtained from our ASM simulations were compared with the X-ray structures. The RMSD of the Cα atoms of the loop with respect to the X-ray structures were calculated along the string nodes of the open and closed loops and represented in Figures S9b and S10b. As suggested from these figures, our ASM simulations successfully drove the loop from one conformation to the other. Upon examining the evolution of the CVs in Figures S9c and S10c, it becomes evident that the conformational changes in both apo and holo states can be decomposed into two stages. During the transition from the closed state to the open state, the hydrogen bond between Tyr308′ and Thr19 must be broken first. The next stage, which corresponds to the sharpest part of the profile, is governed by changes in the Ψ_315′_ and Φ_316′_ torsional angles. The free energy barrier between the closed and open states is thus predominantly dominated by the rotation of this specific peptide group, between residues 315′ and 316′. Finally, the open state is stabilized with the formation of a new hydrogen bond between Tyr308′ and Pro274, along with the formation of a small turn, α helix-like structure (as indicated by the change in the d_3_ distance). This overall behavior is very similar to the one described before for the PTP1B system.^40^ As observed in Figure 7, both apo and holo enzymes present a similar free energy barrier for the Tyr-loop closing (17.9 and 16.7 kcal·mol^–1^, respectively). Our results show that, in the absence of the substrate, the open state is more stable than the closed one by 2.8 kcal·mol^–1^, while in the holo form the closed state is more stable by 1.9 kcal·mol^–1^. This agrees with the fact that the open state is observed in the X-ray structures of the apo form, while the closed state appears for the holo form.^20^

**Figure 7:**
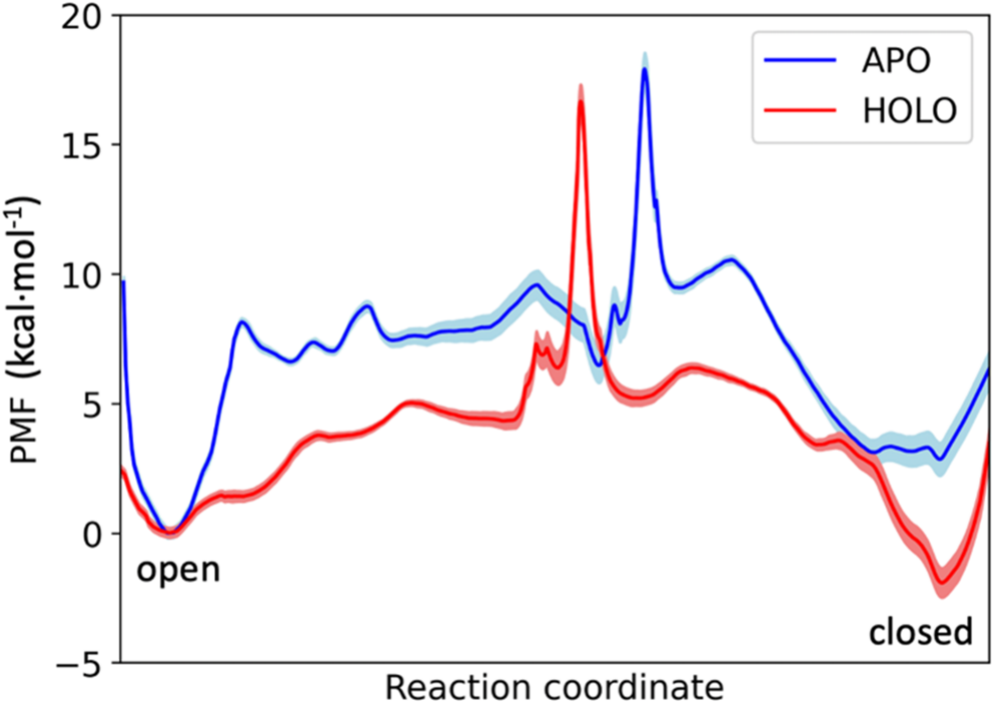
Free energy profiles of the Tyr-loop opening in the apo (blue line) and holo (red line) gpASNase1. The shaded region corresponds to the statistical uncertainty derived by bootstrapping.

To identify the residues responsible for stabilization of the open and closed states in the holo enzyme, we applied the MMGBSA scheme as described before to calculate interaction energies between all of the residues of the loop and each of the remaining residues, treating in this case the whole loop region as the ligand. The list of interaction energies for all residues with absolute values larger than 15 kcal·mol^–1^ is given in Table S7. In Figure 8, we represent those residues that most stabilize the open (red) and closed states (blue color). Residues Leu29, Asp272′, Leu354′, and Met358′ preferably stabilize the closed state of the loop, while Glu155 and Asp152″ (two primes denoting another adjacent monomer) stabilize the open state in gpASNase1. These findings suggest that mutation of these residues might change the conformational equilibrium of the loop and then alter the catalytic properties of gpASNase1.

**Figure 8:**
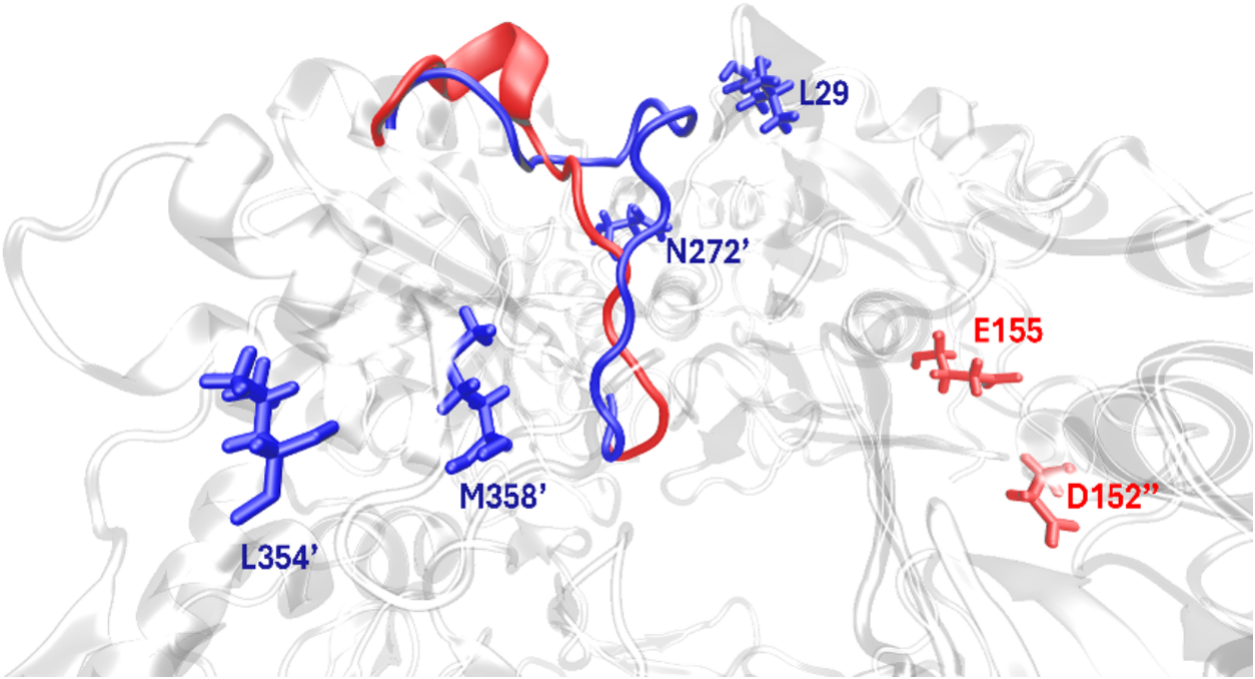
Residues contributing to the stabilization of the open (red color) and closed form (blue color) of the flexible Tyr-loop. Residues with contributions to the absolute interaction energies larger than 15 kcal·mol^–1^ stabilizing the open form of the Tyr-loop are represented in red, while those stabilizing the closed state are given in blue color.

### 3.4 Reaction Mechanism

We investigated the chemical transformation of asparagine to aspartate, taking place in two stages: acyl-enzyme formation and its hydrolysis (see Figure 1). First, we ran QM/MM simulations with the ASM method at the DFTB3D3/MM level of theory and, afterward, the most promising mechanism was recalculated at the B3LYPD3/MM level. Our mechanistic proposal is similar to that proposed previously by Sánchez et al. on the basis of QM/MM optimizations.^7^

The first stage of the chemical reaction catalyzed by gpASNase1 is the formation of the acyl-enzyme (ACE) complex between Asn and Thr19, which requires the elimination of ammonia. Figure 9 shows the reaction scheme for this stage (Figure 9a), together with the CVs used for the exploration of the free energy landscape and definition of the QM subsystem (Figure 9b). The B3LYPD3/MM free energy profile obtained by the string method is depicted in Figure 9c, accompanied by the evolution of CVs (Figure 9d).

**Figure 9:**
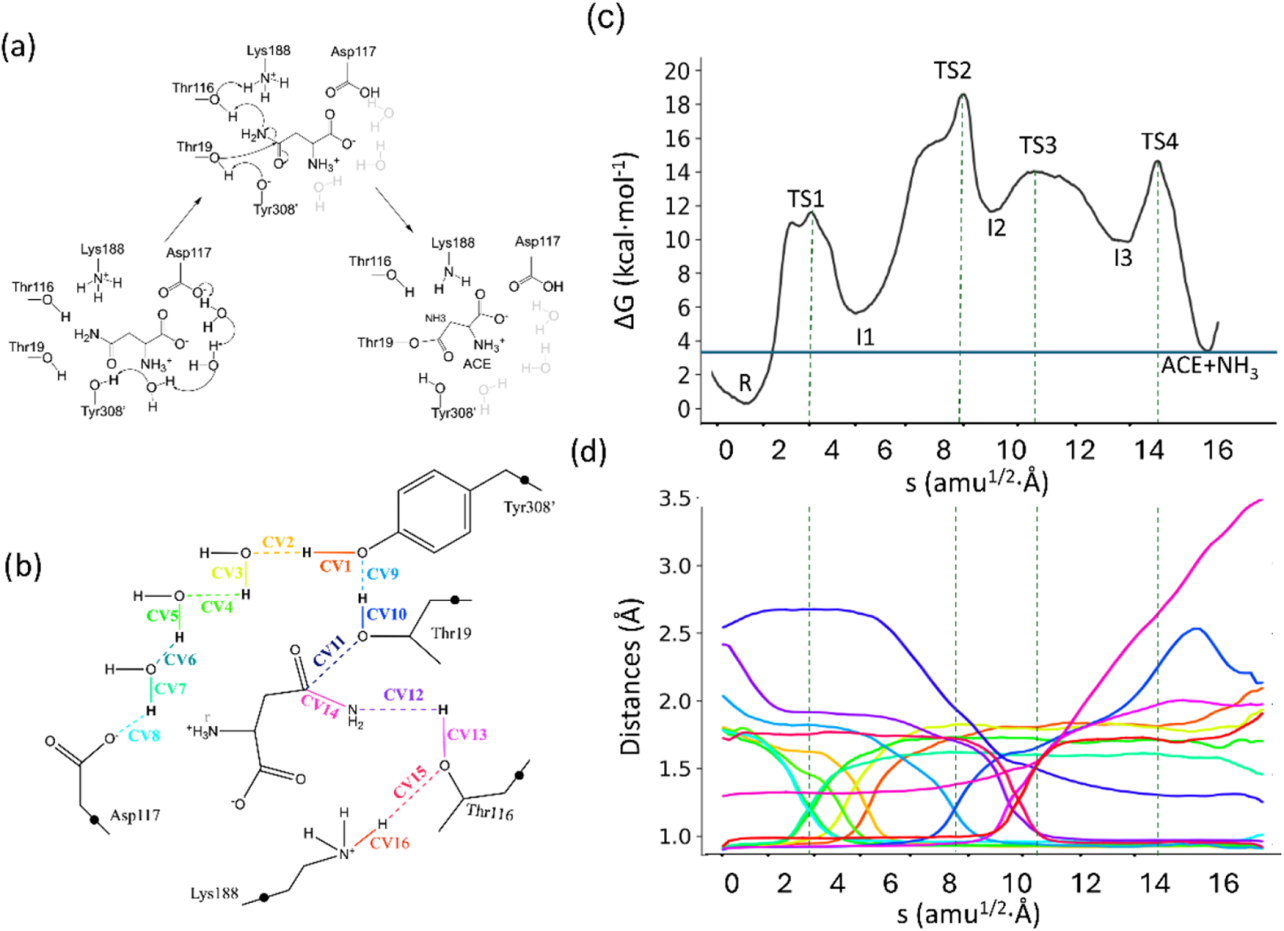
(a) Acylation mechanism in gpASNase1. (b) Representation of the QM region and CVs used to obtain the MFEP. Black dots represent link atoms; (c) Free energy profile along the path-CV (s) obtained at the B3LYPD3/6-31G­(d)/MM level of theory; (d) Evolution of the distances selected as collective variables along the MFEP. The color code corresponds to those used in panel (b). Dashed light-gray lines indicate the position of the transition states.

The mechanism for the acyl-enzyme formation reveals four TSs, labeled as TS1–TS4. First, activation of Tyr308′ involves a proton transfer to Asp117 that takes place through a chain of three water molecules connecting these two residues. This is reflected in the evolution of collective variables CV1–CV8 along the path-CV in Figure 9d. This gives rise to TS1, with an activation free energy of 11.7 ± 0.4 kcal·mol^–1^ with respect to that of the Michaelis complex. This water chain was observed in multiple snapshots of Michaelis complex MD simulations. We confirmed that these waters were also present when the solvent MM model was changed to OPC.^51,52^ Following this, the nucleophilic Thr19 is activated by means of a proton transfer to Tyr308′, as evidenced by the evolution of CV9 and CV10 in Figure 9d. This occurs simultaneously with the nucleophilic attack of Thr19 on the Cγ atom of the substrate (see the evolution of CV11 in Figure 9d). These two events result in TS2, with a relative free energy of 18.7 ± 0.7 kcal·mol^–1^. TS2 is stabilized by the interaction of the carbonyl oxygen atom of Asn with the oxyanion hole residues (Thr19 and Thr116) that provide hydrogen bonds to assist the development of negative charge on this atom during the nucleophilic attack (Figure S11a). In TS3, a proton is transferred from the hydroxyl group of Thr116 to the Nδ atom of the substrate (see the evolution of CV12 and CV13), a process assisted by the proton transfer from Lys188 to Thr116 (see the evolution of CV15 and CV16 in Figure 9d). TS3 presents a relative free energy of 13.7 ± 0.9 kcal·mol^–1^. The last step (TS4, with a free energy of 14.9 ± 0.7 kcal·mol^–1^) consists of breaking the Cγ-Nδ bond (CV14) in the substrate and the release of ammonia. The product state (ACE with NH_3_ still in the active site) exhibits a free energy of 3.9 ± 0.3 kcal·mol^–1^ relative to that of the Michaelis complex.

To complete the enzymatic cycle corresponding to this reaction stage, we estimated the free energy change associated with the release of ammonia from the active site to the bulk. This free energy change was calculated using TI, as detailed in the Supporting Information. The overall free energy computed for elimination of NH_3_ from the active site was −4.2 ± 0.7 kcal·mol^–1^. This value is very similar to those documented for ammonia release in other enzymatic systems.^53^ The observed change in free energy can be explained considering that in aqueous solution, NH_3_ is able to form one hydrogen bond more than in the active site of gpASNase1. Incorporation of the free energy associated with ammonia release into the reaction free energy profile yields a total free energy change for the acylation stage of −0.3 ± 1.1 kcal·mol^–1^.

The second stage of the enzymatic cycle involves the hydrolysis of the ACE complex. Mechanisms involving the participation of different numbers of water molecules and/or different residues were explored at the DFTB3/MM level. Afterward, the mechanism with the lowest activation free energy was reevaluated at the B3LYPD3/MM level. This mechanism is shown in Figure 10a, together with the definition of the QM region and the set of collective variables (CVs) employed for the exploration (Figure 10b). The obtained free energy profile is presented in Figure 10c, complemented by the evolution of the corresponding CVs along the path-CV in Figure 10d. According to these results, the deacylation stage starts with the deprotonation of Thr116 by Lys188 (see CV6 and CV7 in Figure 10d), which was deprotonated during the acylation stage. Almost simultaneously, Thr116 activates the hydrophilic water, subtracting one of its protons, as evidenced by the evolution of CV4 and CV5, as shown in Figure 10d. These proton transfer events take place concomitantly with the nucleophilic attack of a water molecule oxygen atom on the Cγ atom of the substrate, as seen by the decrease in CV3 in Figure 10d. These processes lead to TS5 with an activation free energy of 12.2 ± 0.7 kcal·mol^–1^ relative to that of the ACE complex (see Figure 10c). TS5 is again stabilized by the oxyanion hole residues (Thr19 and Thr116) due to the negative charge developed on the carbonyl oxygen atom of the substrate. Following the nucleophilic attack, a tetrahedral intermediate (I4) is formed with a free energy of 7.6 ± 0.6 kcal·mol^–1^ with respect to the ACE complex. From this intermediate, the reaction proceeds with the proton transfer from Tyr308′ to the acyl oxygen atom, as indicated by the evolution of CV1 and CV2 in Figure 10d, which takes place simultaneously with the breaking of the acyl-enzyme bond, reflected in the increase of the AsnCγ-Thr19Oγ distance (CV8). Finally, the protonation state of Tyr308′ is regenerated by means of a water-mediated proton transfer from Asp117, a residue that was protonated during the acylation stage. This is evidenced by the pairs of CVs describing the proton transfer between these residues (see the evolution of CV9–CV12 in Figure 10d). The resulting transition state (TS6, see Figure S11b) exhibits a free energy of 17.9 ± 0.7 kcal·mol^–1^ relative to the acyl-enzyme complex. The product formed after TS6 is an aspartic acid, protonated in its side chain carboxylic group, a proton that can be easily donated to the solvent after product release. The free energy of this product with respect to the ACE complex is −2.1 kcal/mol, and then, the overall enzymatic process including ammonia release is exergonic, with a reaction free energy of −2.4 ± 1.2 kcal·mol^–1^ relative to the Michaelis Complex.

**Figure 10:**
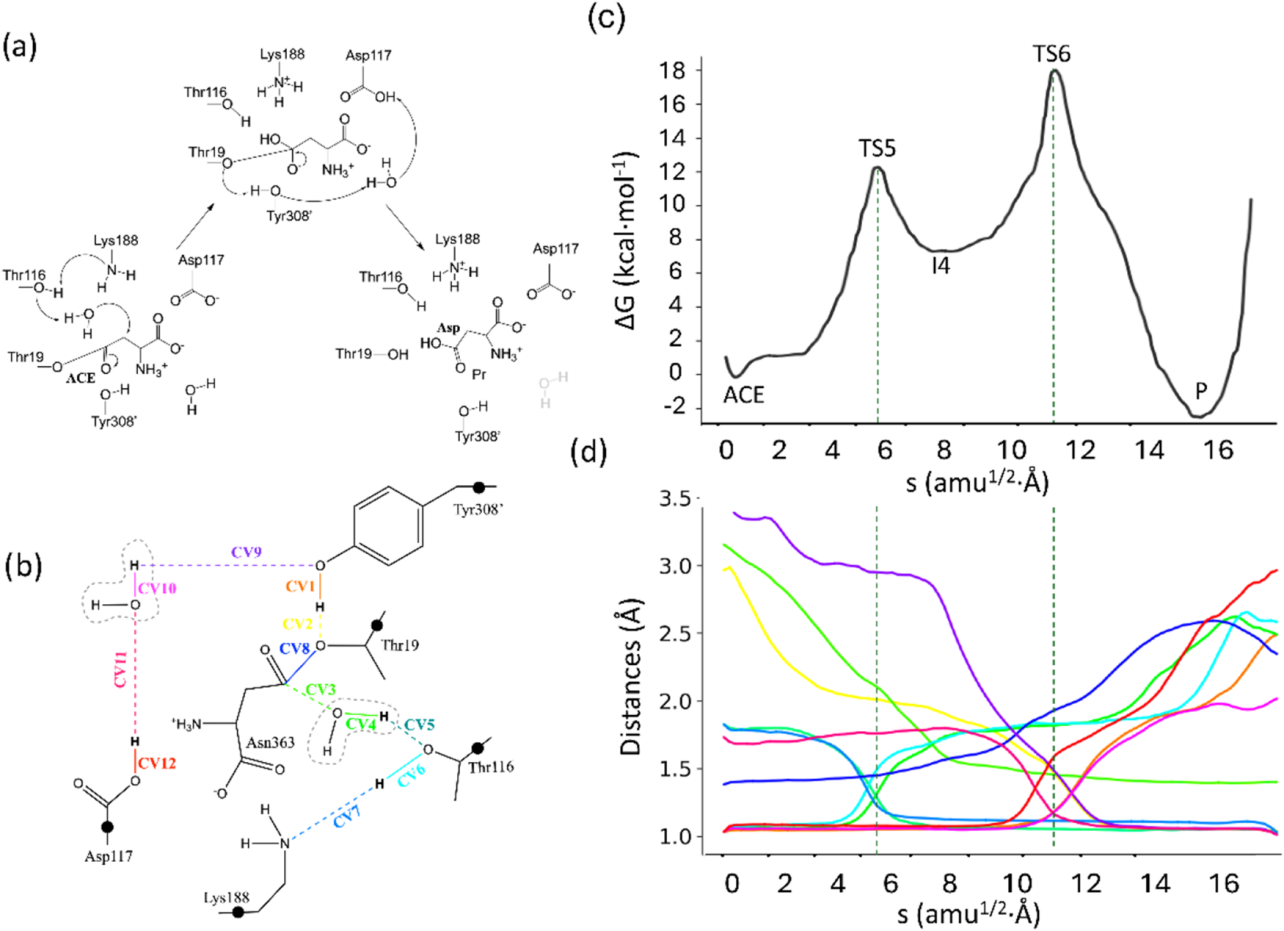
(a) Acyl-enzyme hydrolysis reaction mechanism in gpASNase1; (b) representation of the QM region and CVs used to obtain the MFEP. The black dots represent the link atoms; (c) free energy profile along the path-CV (s) obtained at the B3LYPD3/6-31G­(d)/MM level of theory; and (d) evolution of the distances selected as collective variables along the MFEP. The color code corresponds to that represented in panel (b). Dotted light-gray lines indicate the position of the transition states.

Considering the free energy profile of the whole chemical process (acylation and acyl-enzyme hydrolysis stages), TS2 is the TS corresponding to the rate-limiting step. The corresponding activation free energy (18.7 ± 0.7 kcal·mol^–1^) is in good agreement with the free energy value derived from the experimental *k*_cat_ (16.1 kcal·mol^–1^),^20^ in particular, considering that we have not included in our calculations zero-point energy or tunneling effects, which are expected to decrease the activation free energy.

### 3.5 Electric Field Analysis

In the rate-limiting step (TS2), a negative charge is built on the carbonyl oxygen atom of the substrate. Therefore, the stabilization of this charge seems to be crucial for accelerating the chemical process. So far, only the role of those residues forming the oxyanion hole (Thr116 and Thr19) has been discussed.^7,20^ However, long-range electrostatic effects coming from individual residues or group of residues can also play an important role in catalysis, as previously shown in the case of β-lactamases.^54^ To analyze these possible effects, we computed the electric field created by the environment projected along the carbonyl bond of Asn during a 1 μs trajectory run of the acyl-enzyme state. As depicted in Figure 11a, a positive electric field means an electrostatic contribution favoring the development of a negative charge on the oxygen carbonyl atom and then stabilizing TS2. We evaluated the contributions of individual residues and of structural motifs, summing up the contributions of its residues. The contributions of all those residues with a projected electric field larger, in absolute value, than 2 MV·cm^–1^ are shown in Figure 11b. Finally, the per-motif contribution is depicted in Figure 11c for the tetrameric structure of gpASNase1.

**Figure 11:**
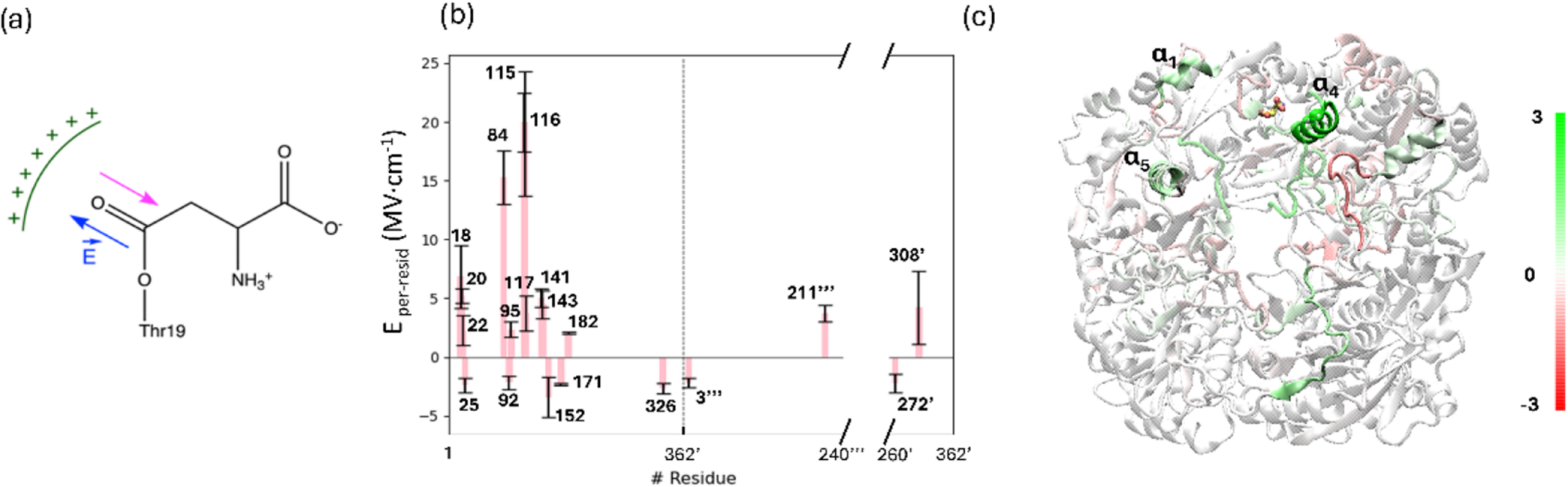
Decomposition of the electric field along the C=O bond of the substrate in the acyl-enzyme state. (a) Schematic representation of the electric field that promotes the transfer of the negative charge from the carbonyl group of the substrate toward the oxygen atom. (b) The values of the projected electric field per residue. Only residues with a contribution larger in absolute value than 2 MV·cm^–1^ are shown. Residues of the adjacent protomer (chain D) are labeled with prime, whereas residues of chain B and chain C are labeled with two and three primes, respectively. Standard errors were calculated as the standard deviation of the mean; (c) Per-motif contribution to the projected electric field for the entire tetrameric gpASNase1 (Color scale units in MV·cm^–1^).

As expected, residues closer to the active site contribute more to the electric field created on the carbonyl bond of the substrate. Among those with a positive contribution, thus stabilizing the reaction TS, are Gly18, Leu20, Met22, Asp84, Gly115, Thr116, Asp117, Gly141, Gln143, Arg182, Asp211″, and Tyr308′. Most of these residues also contribute to the binding of the substrate and discriminate between Asn and Gln substrates (see Figure 4). As evidenced from Figure 11b, negative contributions to the electric field, an electrostatic effect that destabilizes the development of negative charge on the substrate carbonyl oxygen atom, are, in general, significantly smaller and due to a reduced subset of residues Lys25, Asp92, Asp152, Glu171, Glu326, Arg3″, and Asn272′. As seen from Figure 11c, our results suggest that two enzymatic motifs, α_1_ and α_4_, can also contribute to electrostatic stabilization of the TSs during the chemical reaction in gpASNase1. The dipole moments of these two α-helixes create a positive electric field along the carbonyl bond of the substrate. This highlights the importance of selecting an adequate scaffold for the design of new enzymes, as the electric fields are emerging as a critical feature in enzyme catalysis, which may also contribute to improving enzyme design.^55^

### 3.6 Rationalization of the Properties of ASNase Chimeras

As mentioned in the Introduction, one of the possible strategies to overcome the present limitations in ALL treatment is the design of ASNase chimeras with improved immunogenetic properties. In this sense, there is at least one proposal of a humanized version of gpASNase1.^19^ The authors employed DNA shuffling and managed to obtain two chimeras with high sequence similarity to the human enzyme (hASNase1) while still keeping the kinetic and binding properties of gpASNase1. The *K*_M_ values for two chimeras, 63-hC and 65-hC, were 47 and 74 μM, respectively, while the values of the human and guinea pig versions are 3500 and 50 μM, respectively.^19^ The reported catalytic rate constants are 32 and 40 s^–1^ for 63-hC and 65-hC, respectively, while the values found for the human and guinea pig variants are 17 and 41 s^–1^, respectively.^19^

Figure 12 provides an overview of our findings displaying the sequence alignment for the N-terminal domain of gpASNase1, the two chimeras (63-N and 65-N), and hASNase1. Additionally, Figure 12 also includes the results of multiple sequence alignments (MSAs) with the most similar proteins (asparaginase in all cases; see the Supporting Information). In this work, we have identified 72 residues and two structural motifs (α1 and α4) as important for either the binding selectivity, reactivity, or the stabilization of the active form of Tyr-loop in the gpASNase1 variant. These residues are highlighted with red bold letters in Figure 12. Of them, 32 residues are identical in the hASNase1 and gpASNase1 versions. Figure S12 compares the contributions to substrate binding and Tyr-loop conformational change of those residues that are different in both enzymes, showing that those present in the guinea pig version display improved properties. In fact, 63-hC and 65-hC chimeras incorporate up to 65 and 63 of these residues, respectively; which represents 42 and 44% of the total number of mutations incorporated in these designs. In addition, we found that 30 of these residues were preserved in the MSA (blue squares in Figure 12). The positions that were mutated in the chimeras and were found to be preserved in the MSA are Leu20, Ile96, Pro136, Ser191, and Asn318. Apart from these MSA preserved residues, we also identified 27 additional residues important for gpASNase1 activity that were incorporated in both chimeras: Ser7, His10, Lys25, Val36, Thr37, Leu38, Asp91, Asp92, Arg95, Arg98, Glu101, Pro136, Asn177, Gln192, Lys193, Glu195, Tyr216, Asp217, Lys223, Asp226, Gly307, Thr310, Leu340, Glu342, Glu346, Gln349, and Ala353. Lastly, 4 additional residues, identified as important in gpASNase1, were found to be mutated in at least one of the chimeras (Lys48, Arg147, Asn151, and Ala153).

**Figure 12:**
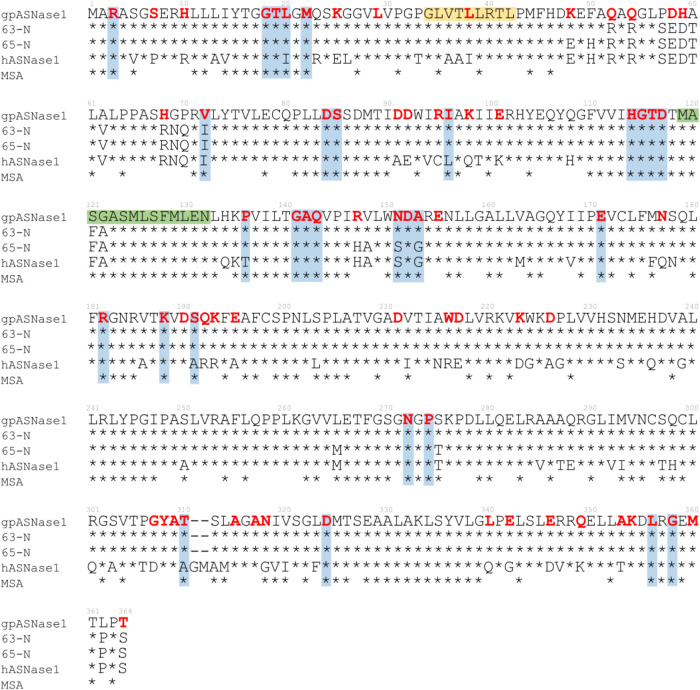
Sequence alignment of the N-terminal domain of gpASNase1, two chimeras (63-N and 65-N), and hASNase1. The result of the MSA presented in this figure includes only the top 45–55% of highly conserved residues preserved in gpASNase1, each marked with an asterisk. gpASNase1 residues identified to have important contributions to binding, loop closing, or electrostatic catalysis are highlighted in red bold letters. Those residues that coincide with the results of MSAs are shaded light blue, while yellow and green bars correspond to the α_1_ and α_4_ structural motifs.

The two chimeras, 63-hC and 65-hC, differ at positions 48, 50, 147, 148, 151, 153, 265, and 275 (Figure 12). In 65-hC, these residues were kept as in hASNase1, while in 63-hC, they were mutated as in gpASNase1. Interestingly, our results suggest that nearly all of these positions form stronger interactions with the substrate in gpASNase1 compared to the corresponding residues in hASNase1 (see Figure S12). This correlates with the lower *K*_M_ value observed at 63-hC, indicating better substrate binding. Our simulations also point out that residues at positions 59 (Asp), 68′, and 68‴ (Arg) display weaker interaction with the substrate when compared to the residues at the same positions in gpASNase1 (His in both cases). Although these residues are far from the active site, they are charged and establish long-range Coulombic repulsive interactions with the substrate, resulting in weaker binding. In the chimeras, these residues were kept as in hASNase1 (Asp and Arg) and then our results suggest that mutating both positions in the chimeras to the residues in gpASNase1 (Asp59His and Arg68His) could improve substrate binding.

Our results also suggest that mutations Arg52Gln, Glu58Asp, Glu59His, and Ile72Val are expected to stabilize the closed form of Tyr-loop (see Figure S12). Interestingly, residue Val72 also appears to be preserved in the MSA (Figure 12) but has not been mutated in either of the chimeras. Regarding the selectivity of the enzymes toward Asn with respect to Gln, our results suggest that no alteration in the selectivity should be expected in the chimeras because the residues that contribute most to this property (Gly18, Met22, Asp84, Ser85, Arg95, His114, Gly115, Asp117, Ala142, and Asp190) are already incorporated in their sequences.

Finally, most of the positions that have been identified to contribute to the electrostatic stabilization of the rate-limiting TS (18, 20, 22, 84, 95, 115, 116, 117, 141, 143, 182, 211, and 308) are the same in gpASNase1 and hASNase1, which could explain their similar kinetic properties. Two structural motifs (α_1_ and α_4_) were identified to produce an electric field that also stabilizes the reaction TS. Interestingly, the entire α_4_ motif seems to be quite preserved in the MSA (see the green bar in Figure 12). Given that the final goal is the clinical use of ASNases in the treatment of ALL, we confirmed that preservation of these motifs in an eventual ASNase design would not cause a strong immunogenic response. As shown in Figure S13 (details in the Supporting Information), neither of the two structural motifs (α_1_ and α_4_) are predicted to display strong binding with the HLADRB1_0701 allele, which is associated with a high risk of hypersensitivity reactions and allergies after treatment with bacterial ASNases.^56^

## 4 Conclusions

In this study, we have conducted classical molecular dynamics (MD) and QM/MM calculations to investigate the catalytic properties of gpASNase1. This enzyme catalyzes the transformation of asparagine into aspartate, showing a large affinity and selectivity for the substrate. After closing the active site loop (Tyr-loop), the enzymatic reaction takes place in two stages: first the formation of an acyl-enzyme complex after the reaction between the substrate and Thr19, and second the hydrolysis of this complex with the formation of aspartate. Analysis of key residues contributing to the different steps of the catalytic cycle (binding, loop closing dynamics, and reactivity) is of interest for the design of new enzymes to be used as enzymatic treatments for acute lymphoblastic leukemia.

Our MD simulations of the Michaelis complex with asparagine in the active site revealed key protein–substrate interactions for the catalytic activity of the enzyme. First, we showed that Lys188 must be protonated in the Michaelis complex, and thus this residue cannot act as the catalytic base activating the nucleophile. Instead, our MD simulations suggest that the nucleophilic Thr19 is activated after proton transfer to Tyr308′. Notably, the proximity between the Cγ of the substrate and hydroxyl oxygen of Thr19 supports its role as a nucleophile, crucial for the formation of the acyl-enzyme complex.

Thermodynamic integration calculations provided a quantitative estimation of the relative binding free energy between asparagine and glutamine, which is consistent with the experimental observation of the lack of glutaminase activity in gpASNase1. Analysis of the per-residue contribution to the interaction energy with asparagine and glutamine highlighted the significant contribution of specific residues in the active site for the preferential binding of asparagine. These key residues interact with the charged carboxylate and amino groups of the zwitterionic substrate, particularly, Asp84, Ser85, Thr116, and Asp117. Additionally, residues Asp190 and Ala142 contribute to interactions with the nonzwitterionic portion of the substrate, further reinforcing asparagine’s preferential binding. We also explored the conformational dynamics of the Tyr-loop, the loop that contains catalytic Tyr308′, responsible for the activation of the nucleophile during the catalytic activity. The closing of this loop is a key step in the catalytic cycle, and we showed how this process is favored after substrate binding. The closed form of the loop is more stable in the holo enzyme, while the open form is preferred in the case of the apo enzyme. Applying an interaction energy analysis, we identified those residues that contribute significantly to stabilizing the open and closed forms of the loop.

We explored the free energy landscape associated with the catalytic mechanism at the B3LYPD3/MM level of theory. According to our simulations, the reaction mechanism begins with the deprotonation of Tyr308′, which transfers a proton to Asp117 mediated by a water chain. Upon activation by Tyr308′, Thr19 performs a nucleophilic attack on the Cγ atom of the substrate. This step is followed by proton transfer from the Lys188/Thr116 pair to the amino group of the substrate, facilitating the release of ammonia. In a second stage, Thr116 activates a water molecule, enabling the hydrolysis of the ACE complex. This leads to a tetrahedral intermediate, followed by bond cleavage and regeneration of the protonation state of Tyr308′ through final water-mediated proton transfer. The rate-limiting step (18.6 kcal·mol^–1^) was identified to be the nucleophilic attack of Thr19 to the substrate, a process that takes place simultaneously to its deprotonation by Tyr308′. Electric field analysis unveiled the role of specific residues (Gly18, Asp84, Thr116, and Tyr308′) and structural motifs (α_1_ and α_4_ helices) in the stabilization of the negative charge developed on the carbonyl oxygen atom of the substrate at the rate-limiting TS, providing an additional understanding of the enzymatic catalytic efficiency.

Our dissection of per-residue contributions to binding, conformational equilibria, and reactivity was then applied to rationalize the properties of chimeras reported by the Lavie research group.^19^ After a comparative analysis of the per-residue contributions between hASNase1 and gpASNase1, we managed to rationalize around 40% of the mutated positions in the chimeras. Most of the mutations introduced in these chimeras were residues that showed improved contributions to the binding and/or to the stabilization of the closed conformation of the Tyr-loop in the guinea pig variant with respect to the human one. Additionally, we identified six additional mutations that could improve the properties of the chimeras (Arg52Gln, Arg54Gln, Glu58Asp, Asp59His, Arg68His, and Ile72Val) and that could be included in future designs. Also, the entire α_4_ helix, which contributes to TS stabilization, seems to be quite preserved according to the results of multiple sequence alignments and is not predicted to produce a high allergenic response.

## Supplementary Material



## Data Availability

A GitHub repository (github.com/emedio/gpASNaseI) contains the input files used in MD simulations. It also includes parameter files (prmtop) of the tetrameric form of the protein with substrate inside the binding site and input files for the string method. GitHub repository also contains input structures for the first step of the reaction mechanism as well as the PDB structures of the rate-limiting transition state (TS2). The repository also contains parameter files of the acyl-enzyme structure and input files for the TUPÃ electric field analysis. Additionally, the repository contains Michaelis complex structures of the gpASnase1 enzyme with the Asn as a substrate, Gln as a substrate, and the hASNase1 enzyme with Asn as a substrate and allosteric effector. Lastly, GitHub also contains structures of the opened and closed Tyr-loop structures of apo and holo gpASNase1 enzymes.

## Notes

Methodological details about MD simulations and about electrical field analysis; information about thermodynamic integration calculations; figures describing thermodynamic cycles followed in the free energy calculation; tables of details of adaptive string method calculations for the conformational change of the Tyr-loop in gpASNase1 and reactivity; figures of RMSD plots of MD simulations of three replicas; distribution plots of the important distances within the active site of the protein; violin plots of distributions of the Φ and Ψ dihedral angles in the open and closed Tyr-loop and two distances obtained from MD simulations of the APO and HOLO forms of gpASNase1; figure of probability distributions of the Ψ_315′_ and Φ_316′_ dihedral angles; figure representations of the free energy change in the closed-to-open conformational change in apo and holo gpASNase1, evolution of the collective variables and RMSD measured along the path-CV with respect to the X-ray structures; table of the interaction energies of the Tyr-flexible loop in the open-loop and closed-loop states with individual residues; transition-state structures of the rate-limiting steps for the acyl-enzyme formation and its hydrolysis; figure of the comparison of differential interaction energies of the substrate and Tyr-loop in hASNase1 and gpASNase1; technical information about multiple sequence alignment (MSA); and figure depicting structural motifs exerting strong binding with the HLA-DRB1*07:01 allele (PDF)
