# Supplementary material for: Unveiling the Catalytic Mechanism and Conformational Dynamics of Guinea Pig l‑Asparaginase Type 1 for Leukemia Drug Design
Source: ACS Catal. 2025 Apr 29;15(10):7919–33. doi: 10.1021/acscatal.4c07791 (PMC12128190; doi:10.1021/acscatal.4c07791)
Supplement: Supplementary file 1 [file cs4c07791_si_001.pdf]

Supporting Information

Unveiling the Catalytic Mechanism and Conformational Dynamics of Guinea Pig L-Asparaginase Type 1 for Leukemia Drug Design

Milorad Andjelkovic[1,2], Kirill Zinovjev[1], Jose Javier Ruiz-Pernía[1]*, Iñaki Tuñón[1]*

[1] Departamento de Química Física, Universidad de Valencia, 46100 Burjassot (Spain)
[2] Instituto de Materiales Avanzados, Universidad Jaume I, 12071 Castelló (Spain)

To whom correspondence should be addressed:
ignacio.tunon@uv.es
j.javier.ruiz@uv.es

Index of Content

The Supporting Information is available free of charge on the ACS Publications website. Detailed technical information about MD simulations (p. S3); Detailed technical information about electrical field analysis (p. S3); Information about Thermodynamic Integration calculations (p. S3-S9); Figures describing thermodynamic cycles followed in the free energy calculations (Figs. S1-S3); Tables of details of ASM calculations for the conformational change of the Tyr-loop in gpASNase1 and reactivity (Tables S4 and S5); Figures of RMSD plots of MD simulations of three replicas (Fig. S4); Distributions plots of the important distances within the active site of the protein (Fig. S5); Violin plot of distributions of the Φ and Ψ dihedral angles in the open and closed Tyr-loop and two distances obtained from MD simulations of the APO and HOLO form of gpASNase1 (Figs. S6 and S7); Figure of the probability distributions of the $\Psi_{315'}$ and $\Phi_{316'}$ dihedral angles (Fig. S8); Figure representations of the free energy change in the closed-to-open conformational change in apo and holo gpASNase1, evolution of the collective variables and RMSD measured along the path-CV with respect to the X-ray structures (Figs. S9 and S10); Table of the Interactions energies of the Tyr-flexible loop in the open-loop and closed-loop states with individual residues (Table S5); Transition states structures of the rate limiting steps for the acyl-enzyme formation and its hydrolysis (Fig. S11). Figure of the comparison of differential interaction energies of substrate and Tyr-loop in the hASNase1 and gpASNase1 (Fig. S12); Technical information about Multiple Sequence Alignment (MSA) (p. S17); Figure depicting structural motifs exerting strong binding with HLA-DRB1*07:01 allele (Fig. S13).

A GitHub repository (github.com/emedio/gpASNaseI) contains the input files used in MD simulations. It also includes structures of the gpASNase1 with substrate inside the binding site and input files for the string method. Input structures for the first step of the reaction mechanism as well as the PDB structures of the rate limiting transition state (TS2). Repository also contains parameter files of the acyl-enzyme structure and input files for the TUPÃ electric field analysis. Additionally, the repository contains Michaelis complex structures of the gpASnase1 enzyme with the Asn as a substrate, Gln as a substrate and hASNase1 enzyme with the Asn as a substrate and allosteric effector. Lastly, GitHub also contains structures of the opened and closed Tyr-loop structures of apo and holo gpASNase1 enzyme.

MD equilibration. Minimization was carried out in 1000 steps, out of which first 20 steps with steepest descent method and then switched to conjugate gradient method. After minimization, final structures were heated to 310 K using Langevin dynamics with a collision frequency equal to 1.0 ps^{-1} and a linear heating ramp, rising the temperature from 100 to 310 K. During the heating simulations, periodic boundary conditions were applied with isotropic position scaling and the time step was f 1 fs. During the minimization and heating, a restraint parabolic potential was applied to all the protein atoms excluding hydrogens with a force constant of 100 $kcal \cdot mol^{-1} \cdot Å^{-2}$. Heated structures were then relaxed under the constant pressure for 1 ns, and then for an additional 1 ns lowering the restrain weigh on the heavy atoms from to 10 $kcal \cdot mol^{-1} \cdot Å^{-2}$. A whole system was minimized again, restraining now only the backbone atoms of the protein, allowing side chains to adapt. Afterwards, a set of short equilibration simulations were run at a constant pressure for 1 ns, each time lowering the restrain weight applied to the backbone atoms for 2 $kcal \cdot mol^{-1} \cdot Å^{-2}$, until reaching restrain free simulation. After releasing all restrains, equilibrated structures entered a production stage of 1000 ns simulations in the NVT ensemble performed in Amber22 GPU version of pmemd.[1,2] The time step was then increased to 2 fs since SHAKE[3] algorithm was applied to constraint bonds involving hydrogen atoms. To achieve better sampling, three replicas of 1 μs were run on each system.

Electric Field Calculations. The analysis of the electric field was done using TUPÃ software.[4] Generated MD trajectory of was used derivate short-range electrostatic interactions between atomic partial charges. These interactions were calculated via Coulomb's Law. The software used calculates the electric field exerted by the environment set on the probe, in this case midpoint of the C=O bond in the substrate. Using this mode, the projection of the electric field on the bond was calculated. Additionally, TUPÃ allows per-residue decomposition of the electric field that gave rise to the results presented.[4] In this work, TUPÃ was employed to examine the electric field in the trajectory of the acyl-enzyme state. The classical MD simulation was run for 1μs as previously described.

Thermodynamic Integration Calculations of the pKa of Ly188. The pK_a of the Lys188 was calculated using the thermodynamic cycle presented in Figure S3. Equation (S1) connects the pK_a shift and the change in the interaction free energy of the N-terminal group in the protonated and unprotonated forms:

$$pK_{prot} = pK_{aq} + \frac{1}{2.303\, kT} \Delta\Delta G \qquad \text{(S1)}$$

In the S1 the $\Delta\Delta G$ represents the difference in free energy change obtained when deprotonating of the Lys in the protein environment and in aqueous solution, $\Delta\Delta G = \Delta G_{B-D} - \Delta G_{A-C}$, k is Boltzmann constant, T is the temperature and pK_{prot} and pK_{aq} stand for the pK values of the terminal group in protein environment and in aqueous solution, respectively. This free energy changes were evaluated from the free energy differences resulting of the alchemical transformations of the neutral into the protonated residue in aqueous solution and in the protein environment (see Figure S1):

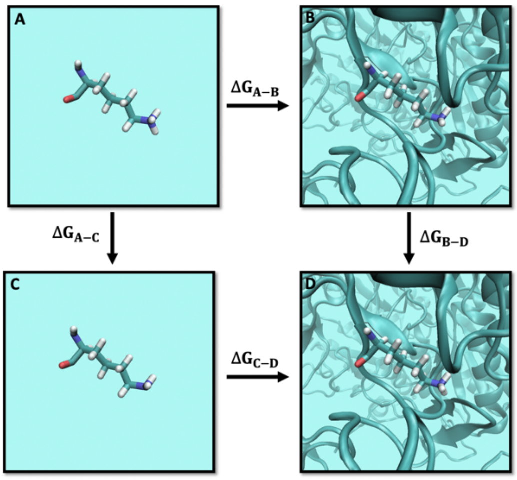

Figure S1. Thermodynamic cycle employed to evaluate the protonation state of Lys, both in aqueous solution and within the protein environment. The blue background indicates the water environment. States A and C represent protonated and deprotonated Lys in water, respectively, while states B and D correspond to protonated and deprotonated threonine within the gpASNase1 enzyme. Alchemical transformations were conducted along the vertical axes A→C and B→D.

The thermodynamic Integration (TI) method was employed as a free energy estimator. The free energy was determined through numerical integration of the derivative of free energy along the coupling parameter (λ) that governs the alchemical transition from an unprotonated to a protonated Lys, as described by equation (S2):

$$\Delta G = \int_0^1 \langle \frac{\partial U(\lambda)}{\partial \lambda} \rangle_\lambda \, d\lambda \tag{S2}$$

Free energy calculations were performed in Amber22[5] using dual-topology approach and following a recently published protocol for GPU-based simulations.[6] Briefly, the temperature was maintained at 310 K using Langevin dynamics with a collision frequency of 1 ps^{-1}. The pressure was kept constant at 1.01325 bar using a Monte Carlo barostat with a pressure relaxation time of 2.0 ps. The unique atom undergoing the alchemical transformation (the additional proton) was placed in the soft-core region for both van der Waals and electrostatic interactions.

All simulations were performed using pmemd.cuda on GPUs, with a time step of 2 fs using SHAKE. The energy was calculated in five replicas to ensure reliable averaged estimation (refer to Table S1). The alchemical transformation was performed using twelve different λ values, employing the Gaussian quadrature method. Each replica ran for 40 ns for each λ value, with the first 5 ns of each simulation designated as an equilibration step at the respective λ window. Subsequently, the five free energy changes derived from the corresponding replicas were average. This average value, representing the free energy change during the alchemical transformation of the residue in water, was then subtracted from the similarly obtained and averaged value within the protein environment. This difference (ΔΔG) was used in the equation (S1). The error was computed as the standard error of the mean (SEM), obtained by dividing the standard deviation (STD) by the square root of the number of replicas.

Table S1. Results of five replicas performed to calculate the free energy change (in kcal·mol^{-1}) associated to the alchemical transformations between the unprotonated and protonated forms of Lys performed in aqueous and protein environment. Average values are given with the corresponding standard deviations.

environment	replica	$\Delta G_{A\text{-}C}$	environment	replica	$\Delta G_{B\text{-}D}$
	1	-4.48		1	2.23
	2	-4.48	holo	2	3.15
aqueous	3	-4.46	protein	3	3.16
	4	-4.46		4	3.31
	5	-4.48		5	3.74
mean		-4.48	mean		3.12
std		0.02	std		0.99

Table S2. Free energies for the alchemical transformation of Lys protonation state with respect to the water, pK_a shifts and pK_a values of the Lys residue in the gpASNase1. The free energy cost associated to the deprotonation of the lysine residue in protein are given at the pH=7.5 and T=310 K. Calculated probabilities of the protonated and deprotonated form of lysine residue are also given at the same pH and temperature.

Form	gpASNase1
$\Delta\Delta G = \Delta G_{B-D} - \Delta G_{A-C}$ (kcal·mol^{-1})	7.6 ± 0.5
$pK_{prot} - pK_{aq}$	5.36 ± 0.5
pK_{prot}	15.83 ± 0.5
$\Delta G(deprotonation)_{pH=7.5,T=310\ K}$ (kcal·mol^{-1})	11.85 ± 0.7
$P(protonated)_{pH=7.5,T=310\ K}$	0.99
$P(deprotonated)_{pH=7.5,T=310\ K}$	0.01

Thermodynamic Integration Calculations of NH_3 leaving the active site. The free energy change (-ΔG_{bind}) associated with NH_3 leaving from the active site can be determined by integrating various factors outlined in the thermodynamic cycle illustrated in Figure S2:

$$-\Delta G_{bind} = \Delta G^{*}_{bulk} + \Delta G^{V^{o}\rightarrow rest}_{decoupled} - \Delta G^{*}_{site} \qquad \text{(S3)}$$

The first (ΔG^{*}_{bulk}) and last (ΔG^{*}_{site}) terms stand for the energy changes of the NH_3 appearance in the bulk water decoupling NH3 in the bound state, respectively. Both terms were derived from an average of five independent replicas (refer to Table S3). Softcore potential was used for the entire NH_3 molecule and the timestep was set to 2 fs using SHAKE. Hamiltonian replica exchange was employed between windows to enhance sampling. The simulation of each window was run for 40 nanoseconds, out of which first 5 ns were considered as equilibration and were therefore not taken into account. To maintain a constant volume for replica exchange between different λ windows, production simulations were conducted under the NVT ensemble. Throughout all stages of the alchemical transformation, NH_3 was kept within the active site by applying a simple restraining potential to the distance between the NH_3 nitrogen and the C_γ od the substrate. The free energy related to restraining the decoupled ammonia (($\Delta G^{V^{o}\rightarrow rest}_{decoupled}$) was computed as:

$$\Delta G^{V^{o}\rightarrow rest}_{decoupled} = -RTln\left(\frac{Q}{V^{o}}\right) \qquad \text{(S4)}$$

where Q is given as:

$$Q = \int_{0}^{\infty} 4\pi r^{2}\, e^{-\beta U_{rest}(r)} dr \qquad \text{(S5)}$$

The force constant of the applied semi-parabolical restraining potential of the form $U_{rest} = k(r - r_0)^2$ was *50* kcal·mol^{-1}·Å^{-2}, where r stands for the AsnC_γ-AsnN_δ distance. The limiting distance of the restraining potential (r_0) was set to 3.5 Å which corresponds to the distance measured in the last node of the string calculation.

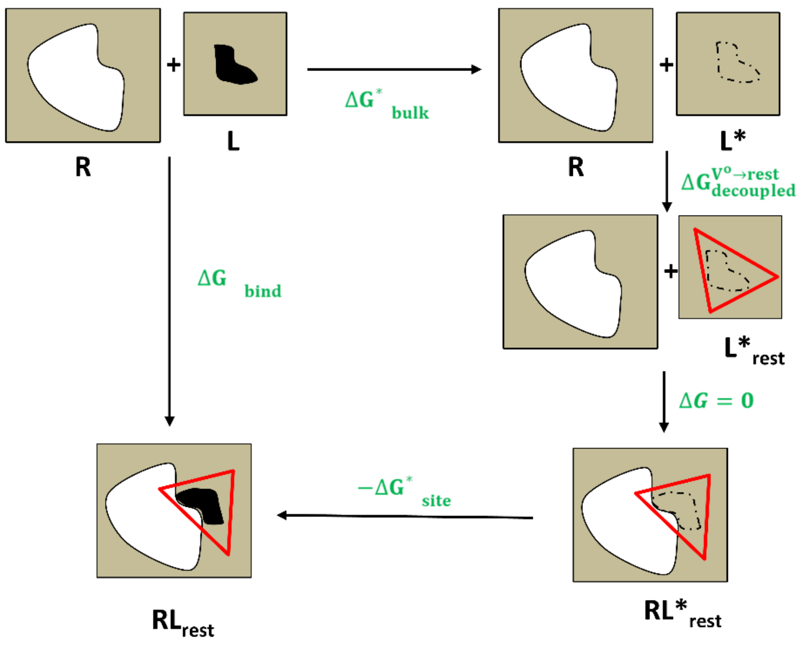

Figure S2. Thermodynamic cycle used to evaluate free energy change of NH_3 leaving the active site. R: unbound protein. L: unbound NH_3 in water. L*: decoupled NH3. RL: protein-NH_3 complex.

When evaluating a standard binding free energy, it's important to incorporate an additional correction accounting for the release of restraints on the coupled NH_3 within the active site ($\Delta G_{coupled}^{rest\rightarrow site}$).[7,8] However, given that the free energy changes we're analyzing refer to the release of a restrained NH_3, as obtained from the string calculation, this particular term was excluded. Considering all factors, the calculated correction was calculated to be 2.74 kcal·mol^{-1}. The term ΔG_{bulk}^{*} is determined to be 4.02 ± 0.07 kcal·mol^{-1}, while ΔG_{site}^{*} is -6.85 ± 0.65 kcal·mol^{-1}. Consequently, the overall free energy associated with NH_3 leaving the active site is found to be -5.57 ± 0.72 kcal·mol^{-1}.

Table S3. Free energy changes associated to alchemical transformations performed in water and protein environments for the release of ammonia from the active site of gpASNase1 to the bulk. Free energy values (in kcal·mol^{-1}) were estimated using TI and each average value is given with the corresponding standard deviation.

Environment	replica	ΔG_{bulk}^{*}	environment	replica	ΔG_{site}^{*}
	1	2.00		1	-2.93
	2	2.01		2	-3.16
aqueous	3	2.02	protein	3	-3.78
	4	2.00		4	-3.62
	5	2.01		5	-3.64
mean		2.01	mean		-3.42
std		0.07	std		0.65

Thermodynamic Integration Calculations of the Relative Binding Free Energy between Ans and Gln. Relative binding free energy between Gln and Asn was calculated similarly to the scheme used to determine ΔG^*_{bulk} and ΔG^*_{site} of NH_3 described before. The schematic thermodynamic cycle followed is given in the Figure S3.

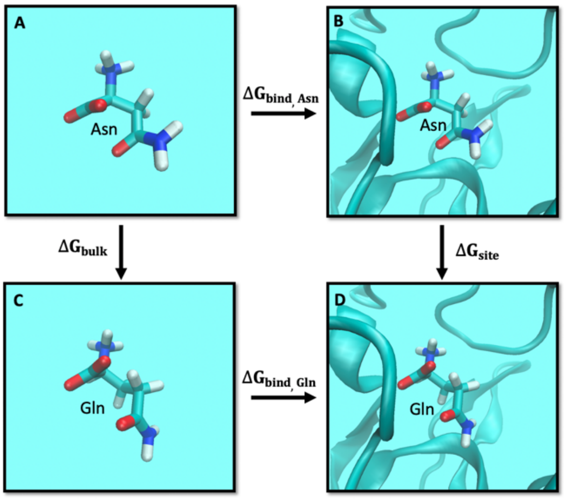

Figure S3. Thermodynamic cycle employed to evaluate the relative binding free energy between Asn and Gln to the gpASNase1. The transformation was performed in the aqueous solution and within the protein environment. The blue background indicates the water environment. States A and C represent Asn and Gln in water, respectively, while states B and D correspond to Asn and Gln in the gpASNase1 enzyme. Alchemical transformations were conducted along the vertical axes A→C and B→D.

Both substrates, Gln and Asn, were treated with the softcore potential along the alchemical transformation. The only difference from the before described procedure is that number of λ windows was increased to 20, in order to reach better overlap between the neighboring windows. The average value, representing the free energy change during the alchemical transformation of the Asn to Gln in water, was then subtracted from the similarly obtained averaged value in the active site of the gpASNase1. This difference (ΔΔG) directly represents the relative binding free energy of Asn and Gln as given in the equation S6:

$$\Delta\Delta G_{bind} = \Delta G_{bind,Asn} - \Delta G_{bind,Gln} = \Delta G_{bulk} - \Delta G_{site} \qquad \text{(S6)}$$

The results of five replicas ion water and active site are given in the Table S4. The relative binding energy is therefore estimated to be $\Delta\Delta G_{bind}$= -6.3 ± 0.6 kcal·mol^{-1}.

Table S4. Free energy changes associated to alchemical transformations of Asn to Gln performed in water ($\Delta G^{*}_{\mathrm{bulk}}$) and the active site of gpASNase1 ($\Delta G^{*}_{\mathrm{site}}$). Free energy values (in kcal·mol^{-1}) were estimated using TI and each average value is given with the corresponding standard deviation.

Environment	replica	ΔG_{bulk}	environment	replica	ΔG_{site}
aqueous	1	-32.78	protein	1	-26.44
	2	-33.00		2	-27.29
	3	-33.05		3	-26.52
	4	-32.75		4	-26.23
	5	-32.56		5	-26.08
mean		-32.83	mean		-26.51
std		0.36	std		0.94

Table S5. Details of ASM calculations for the conformational change of the Tyr-loop in gpASNase1

	APO	HOLO
String nodes	88	88
Number of CVs	5	5
REX period (fs)	500	500
String friction	1000	1000
Force friction	50	50
Preparation (ps)	10	10
String optimization (ns)	30	30
Umbrella Sampling (ns)	10	10
Timestep (fs)	2	2

Table S6. Details of ASM calculations for the reaction mechanism in gpASNase1

	Acyl-enzyme formation	Hydrolysis of the acyl-enzyme
String nodes	96	96
Number of CVs	15	12
REX period (fs)	50	50
Preparation (ps)	0	0
String optimization (ps)	4	4
Umbrella Sampling (ps)	10	10
Timestep (fs)	1	1
Cutoff for DFT/MM interactions (Å)	15	15

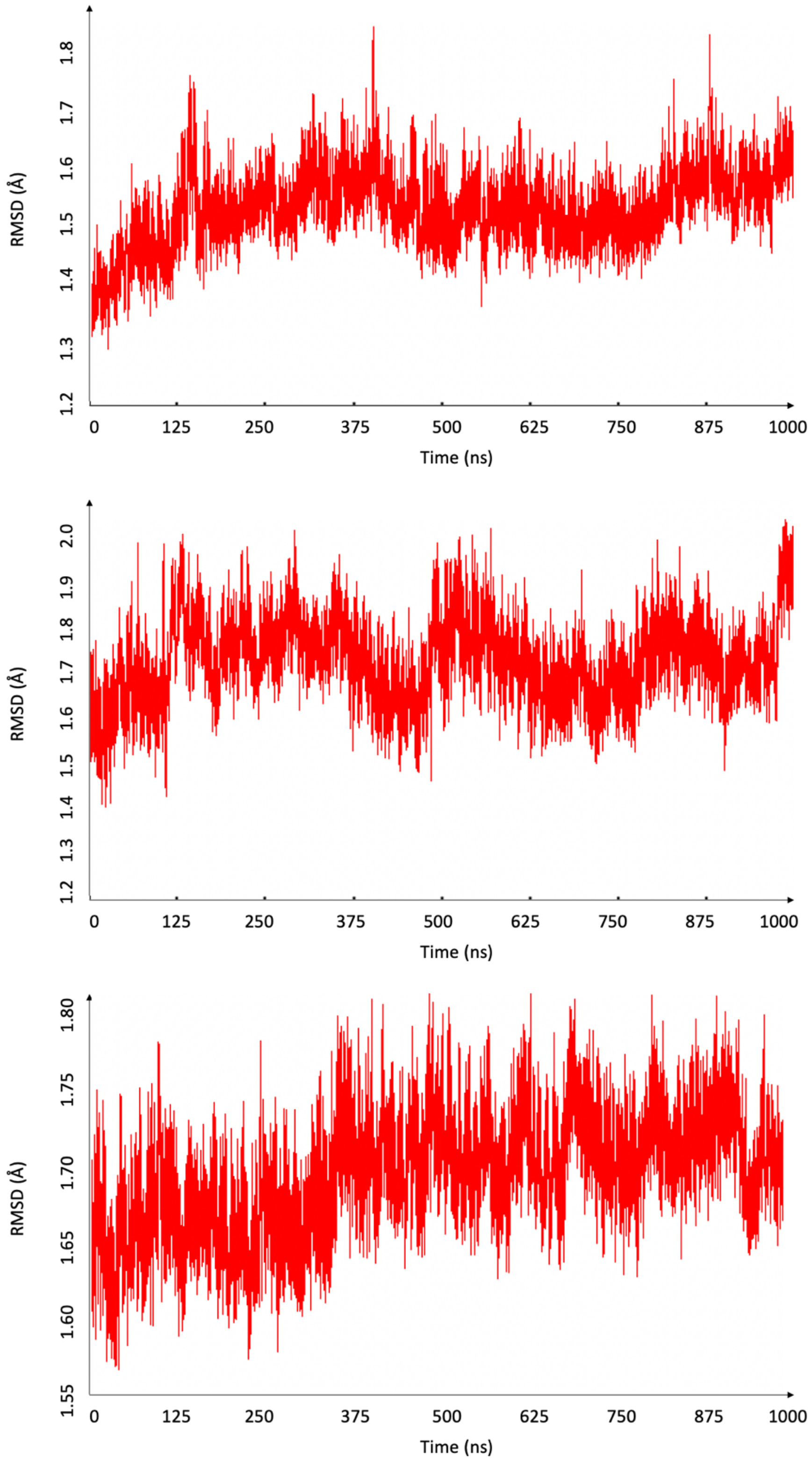

Figure S4. Root mean square deviation along the MD simulations (three replicas) of the Michaelis complex of tetrameric form of gpASNase1 with substrate in the active site.

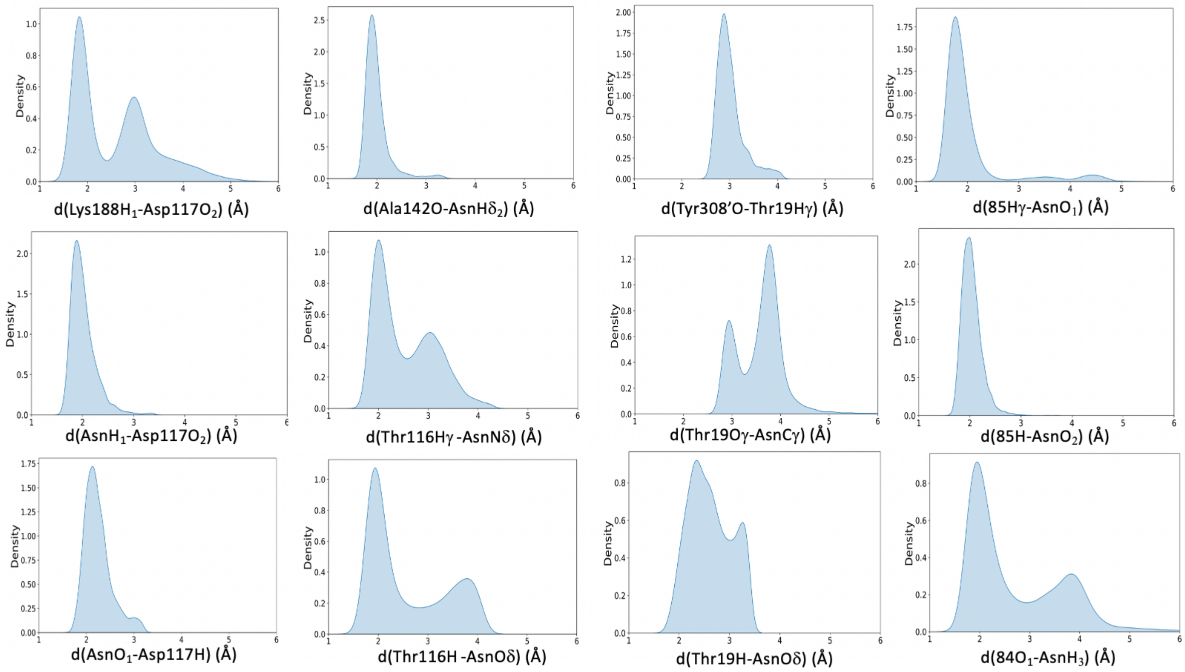

Figure S5. Distributions of the important distances in (Å) obtained over three replicas of 1 μs of classical molecular dynamic simulation run on the Michaelis complex of Asn substrate in the active site of the gpASNase1.

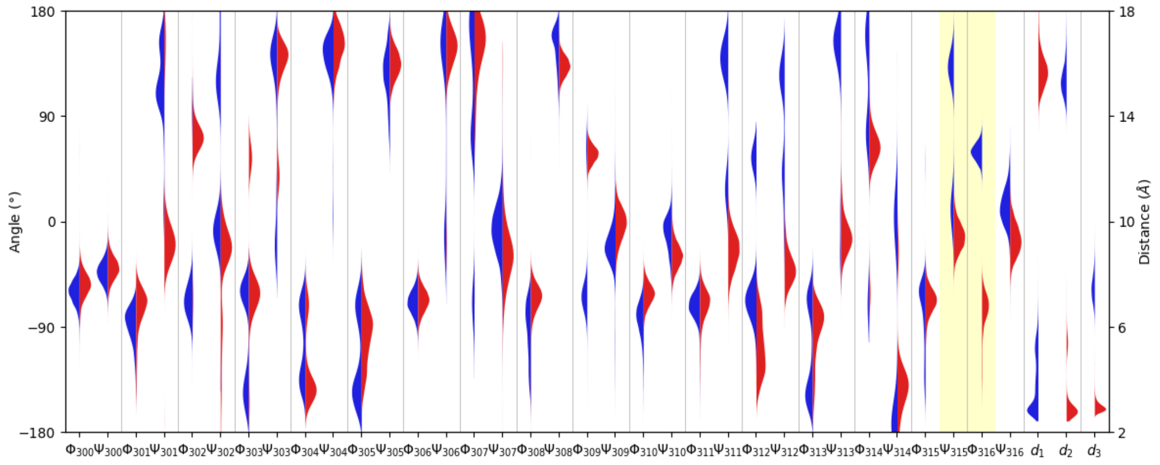

Figure S6. Violin plot of distributions of the Φ and Ψ (in ∘) dihedral angles in the open (red) and closed loop as w
as the two distances obtained from MD simulations of the APO form of gpASNase1. The d_1 is the distance betwe
Tyr308'O_γ and Thr19O_γ, and d_2 is the distance between Tyr308'-O_γ and Pro260O_γ. The distributions with a yell
patched background are the ones chosen to determine the difference between open and closed loop state.

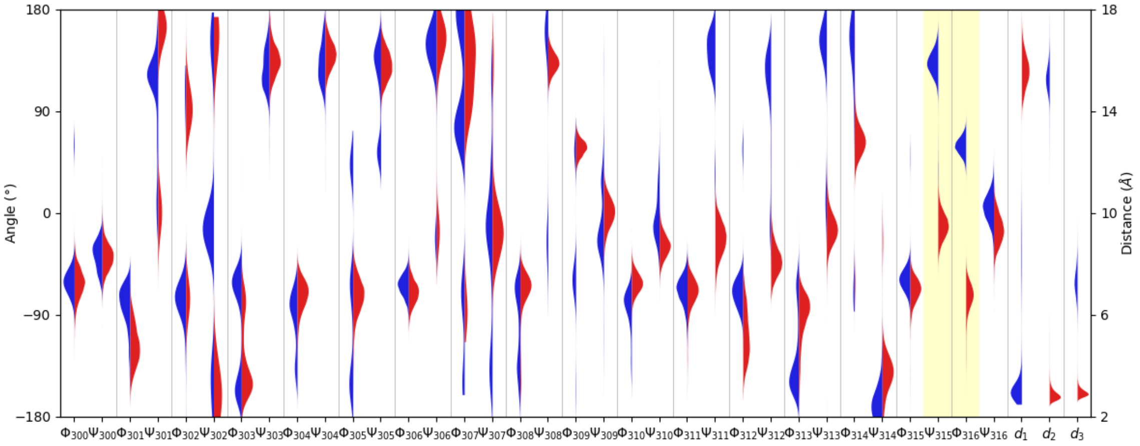

Figure S7. Violin plot of distributions of the Φ and Ψ (in ◦) dihedral angles in the open (red) and closed loop as well as the two distances obtained from MD simulations of the HOLO form of gpASNase1. d_1 is the distance between Tyr308'$O_γ$ and Thr19$O_γ$, and d_2 is the distance between Tyr308'-$O_γ$ and Pro260$O_γ$. The distributions with a yellow patched background are the ones chosen to determine the difference between open and closed loop state.

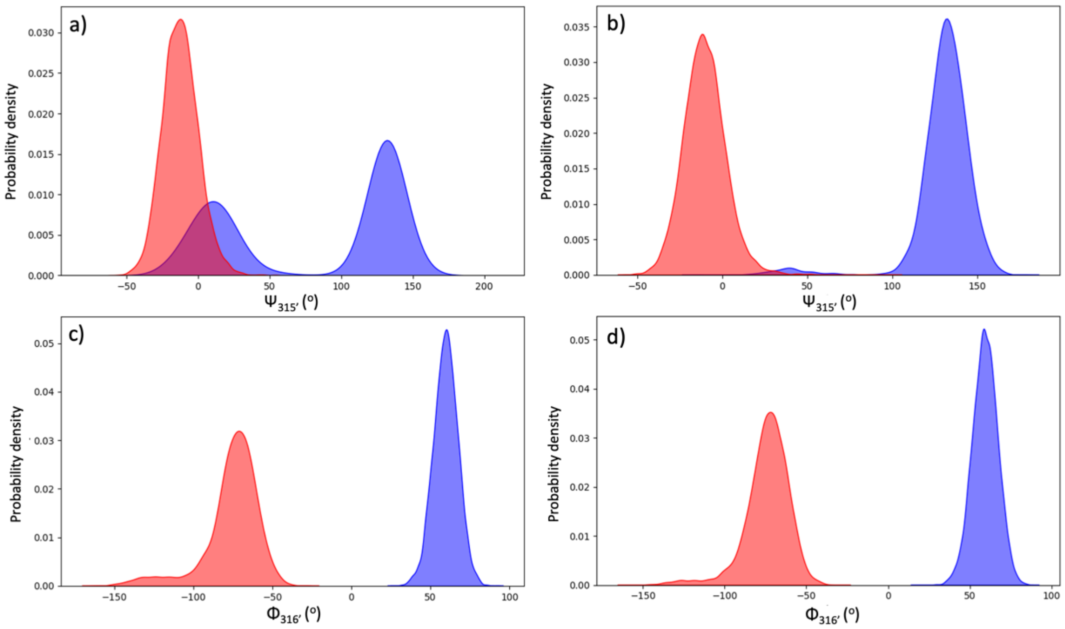

Figure S8. Probability density distributions of the dihedral angles $Ψ_{315'}$ and $Φ_{316}$ (in degrees) in the open (red) and closed loop from MD simulations of the APO form of gpASNase1 (panels a and c) and HOLO form of gpASNase1 (panels b and d).

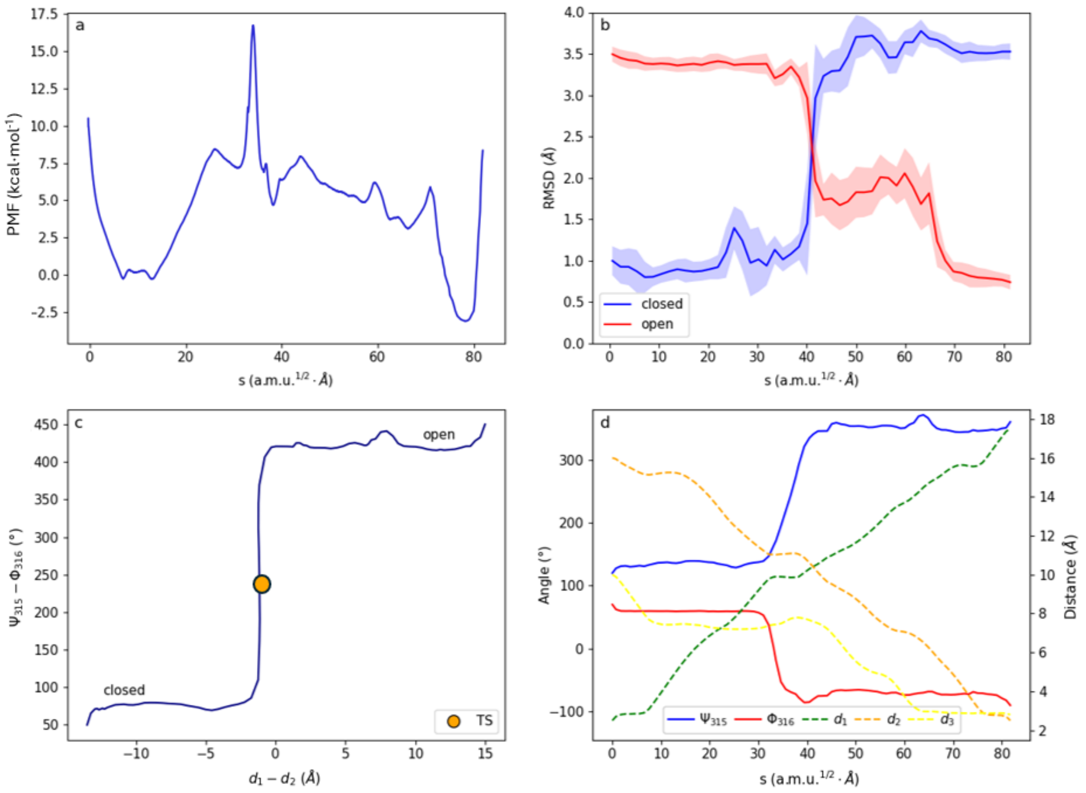

Figure S9. Free energy change in the closed-to-open conformational change in apo gpASNase1. a) Free energy profile for the closed (left) to open (right) transition along the *s* path-CV. b) Average RMSD measured for the C_α atoms of the Tyr-loop for snapshots obtained from the Umbrella Sampling simulations along the path-CV with respect to the X-ray structures corresponding to the closed (blue) and open (red) states. The shaded region corresponds to the statistical uncertainty (95% confidence interval); c) Projection of the MFEP along the antisymmetric combinations of the two distances and two dihedral angles used as CVs. The yellow dot indicates the position of the Transition State; d) Evolution of the individual CVs (distances on the right vertical axis and dihedrals on the left vertical axis) along the MFEP. The CVs used in the ASM calculations are: $\Psi_{315'}$ and $\Phi_{316'}$ torsional angles and the distances Tyr308'O_γ-Thr19O_γ (d_1), Tyr308'O_γ-Pro274O_γ (d_2) and Ala309'C-Ala313'N (d_3).

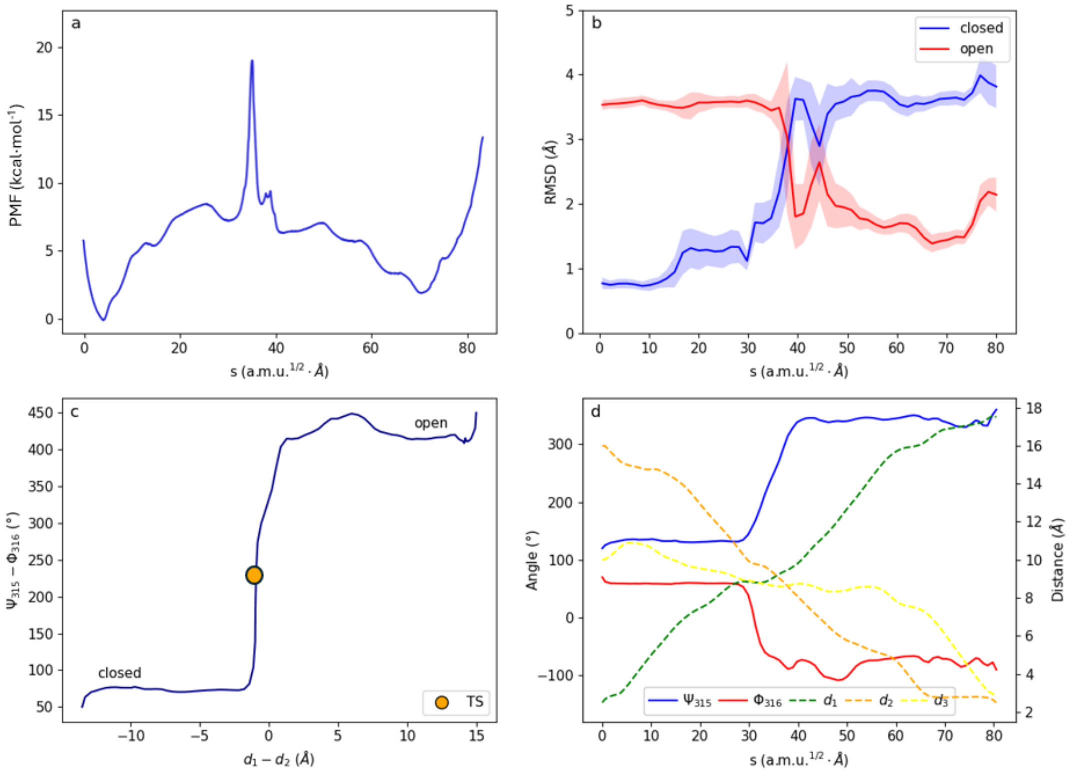

Figure S10. Free energy change in the closed-to-open conformational change in holo gpASNase1. a) Free energy profile for the closed (left) to open (right) transition along the *s* path-CV. b) Average RMSD measured for the C_α atoms of the Tyr-loop for snapshots obtained from the Umbrella Sampling simulations along the path-CV with respect to the X-ray structures corresponding to the closed (blue) and open (red) states. The shaded region corresponds to the statistical uncertainty (95% confidence interval); c) Projection of the MFEP along the antisymmetric combinations of the two distances and two dihedral angles used as CVs. The yellow dot indicates the position of the Transition State; d) Evolution of the individual CVs (distances on the right vertical axis and dihedrals on the left vertical axis) along the MFEP The CVs used in the ASM calculations are: $\Psi_{315'}$ and $\Phi_{316'}$ torsional angles and the distances Tyr308'O_γ-Thr19O_γ (d_1), Tyr308'O_γ-Pro274O_γ (d_2) and Ala309'C-Ala313'N (d_3).

Table S7. Interactions energies (in kcal/mol) with the Tyr-flexible loop in the open-loop and closed-loop states. Only residues having a contribution < -15 kcal/mol are represented. The colored residues correspond to the residue representations in Fig. 8 of the paper contributing only to the open (blue color) and closed (red color) loop stabilization.

Residue	Closed loop	Open loop
**Leu29**	-16.5	
Asp84	-38.8	-18.8
Asp87	-18.2	-17.4
Asp117	-26.1	-26.0
Asp152	-23.1	-24.2
**Glu155**		-16.4
Asp190	-95.3	-84.1
Glu195	-43.	-66.3
Glu266	-18.4	-18.4
Asp322	-15.2	-17.3
Glu326	-21.1	-21.7
**Asp152''**		-16.1
Glu155''	-16.5	-17.6
Asp190''	-15.4	-19.2
Glu195''	-22.4	-30.7
Asp211''	-22.4	-21.8
Asp217''	-17.2	-16.5
Asp190'	-19.5	-23.4
Glu195'	-20.9	-25.5
Glu266'	-19.5	-17.7
**Asn272'**	-20.4	
Gln298'	-59.7	-60.5
Val318'	-81.3	-80.5
Met323'	-34.3	-26.3
Ala327'	-19.3	-16.0
**Leu354'**	-15.1	
**Met358'**	-17.2	

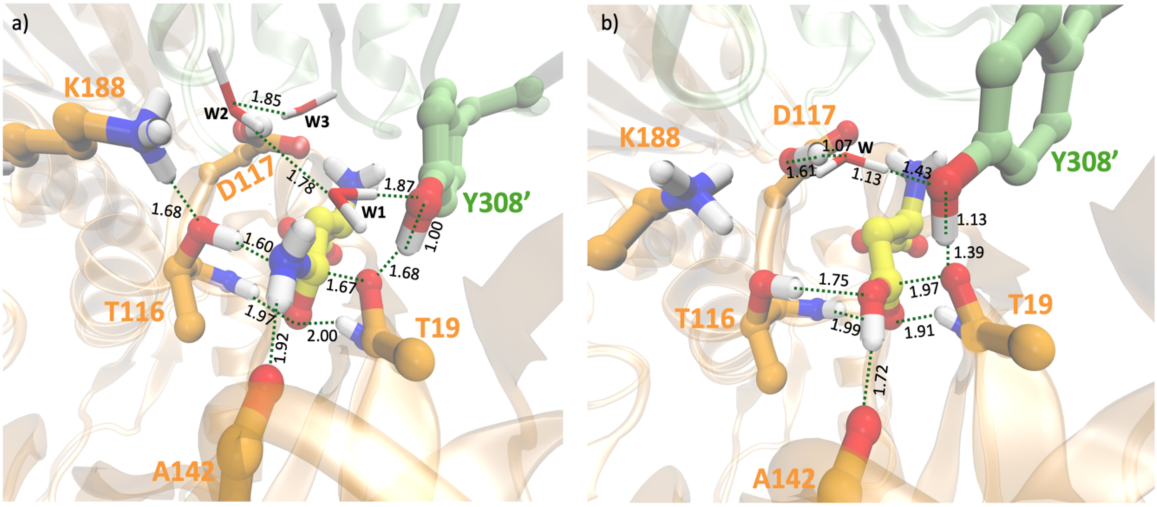

Figure S11. Transition states structures along with some important distances for the formation of the acyl-enzyme complex and its hydrolysis in gpASNase1. (a) TS2 and (b) TS6. All distances are given in Å.

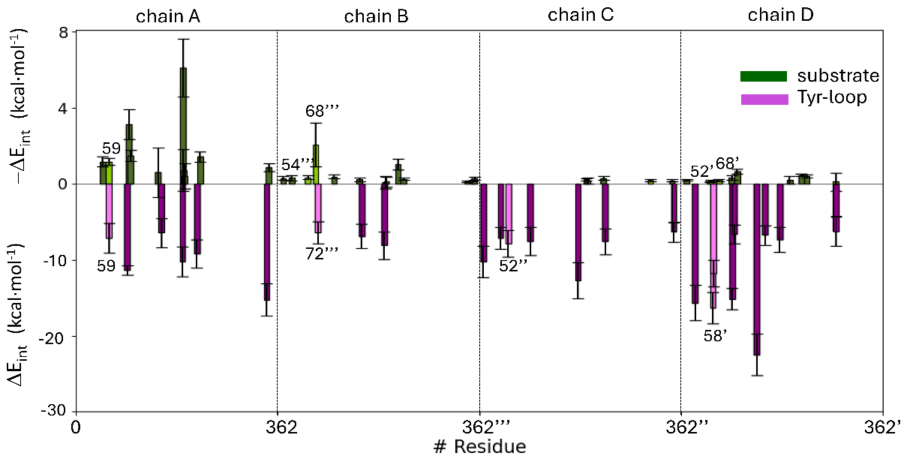

Figure S12. Comparison of differential interaction energies in hASNase1 and gpASNase1. We prepared a model of the Michaelis complex of hASNase1 with Asn and run classical MD simulations following the same procedure as in gpASNase1. The initial structure of hASNase1 was obtained using AlphaFold2 and the substrate was placed in the active sites replicating the main interactions observed in the active site of gpASNase1.The green histogram bars represent the difference in average interaction energy of the substrate with each residue in the human enzyme and each residue in the guinea pig enzyme ($-(E_{Asn\text{-}h,i} - E_{Asn\text{-}gp,i})$). A negative value indicates a preferential interaction with the residue present in guinea pig version. The magenta histogram bars illustrate the difference in average interaction energy of the closed Tyr-loop with each residue in the human enzyme compared to the guinea pig enzyme ($E_{int,\ loop\text{-}gp} - E_{int,\ loop\text{-}h}$). A positive value indicates a preferential interaction of the closed form of the loop with the residue present in guinea pig version. Only residues with $E_{int,Asn}$ values below -0.1 kcal·mol^{-1} and $E_{int,loop}$ values below -10 kcal·mol^{-1} are shown. Residues from the adjacent protomer (chain D) are labelled with a prime symbol, while residues from chains B and C are labelled with double and triple primes, respectively. Darker-coloured histograms indicate residues included in the chimeras designed by directed evolution. Energy values are expressed in kcal·mol^{-1}, and error bars represent standard deviations of the mean values.

Multiple Sequence Alignment (MSA). To create the MSA, four rounds of HHblits searches[9] were carried out against the UniRef30 database (accessed on July 15, 2024) using E-value thresholds of 1e-50, 1e-30, 1e-10, and 1e-4. For each position in the sequence alignment, we calculated the occurrence frequency of each amino acid and identified the most conserved amino acid at each site. We then filtered each position based on the conservation level of the most frequent amino acid and selected approximately the top 50% of the most conserved sites.

Prediction of Epitopes in T-Cells and Determination of Epitopes Density

The prediction of gpASNase1 segments that could bind to the Major histocompatibility complex class II (MHC II) molecules were determined using the NetMHCIIPan 4.1 EL (www.services.healthtech.dtu.dk/services/NetMHCIIpan-4.1/). This tool is a neural network model that predicts the MHC binding values from an amino acid sequence, based on a training set of peptide-MHC class II quantitative binding data covering thousands of human MHC molecules. We selected the HLADRB1_0701 allele, as it is being commonly associated with a high risk of hypersensitivity reactions and a higher risk of allergies after treatment with bacterial ASNase.[10] The prediction of ASNase allergenic peptides from gpASNase1 for the HLA-DRB1_0701 is represented in Figure S13. Peptides predicted as immunogenic epitopes were linearly established by percentile < 10 ("Strong binder (SB)").

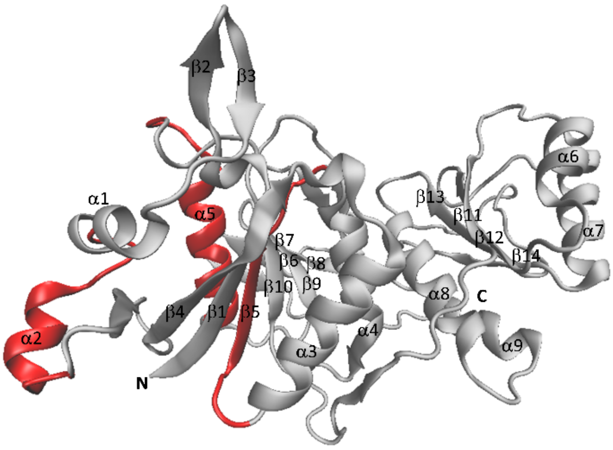

Figure S13. Structural motifs in the monomeric gpASNase1 exerting strong binding with HLA-DRB1*07:01 allele. Grey zones represent zones with no binding with HLA-DRB1*07:01 allele.

References

(1) Le Grand, S.; Götz, A. W.; Walker, R. C. SPFP: Speed without Compromise - A Mixed Precision Model for GPU Accelerated Molecular Dynamics Simulations. *Comput. Phys. Commun.* **2013**, *184* (2), 374–380. https://doi.org/10.1016/j.cpc.2012.09.022.

(2) Salomon-Ferrer, R.; Götz, A. W.; Poole, D.; Le Grand, S.; Walker, R. C. Routine Microsecond Molecular Dynamics Simulations with AMBER on GPUs. 2. Explicit Solvent Particle Mesh Ewald. *J. Chem. Theory Comput.* **2013**, *9* (9), 3878–3888. https://doi.org/10.1021/ct400314y.

(3) Ryckaert, J.-P.; Ciccotti, G.; Berendsen, H. J. C. Numerical Integration of the Cartesian Equations of Motion of a System with Constraints: Molecular Dynamics of n-Alkanes. *J. Comput. Phys.* **1977**, *23* (3), 327–341. https://doi.org/10.1016/0021-9991(77)90098-5.

(4) Polêto, M. D.; Lemkul, J. A. TUPÃ: Electric Field Analyses for Molecular Simulations. *J. Comp. Chem.* **2022**, *43* (16), 1113–1119. https://doi.org/10.1002/jcc.26873.

(5) Case, D. A.; Ben-Shalom, I. Y.; Brozell, S. R.; Cerutti, D. S.; Cheatham III, T. E.; Cruzeiro, V. W. D.; Darden, T. A.; Duke, R. E.; Ghoreishi, D.; Gilson, H.; Gohlke, H.; Goetz, A. W.; Greene, D.; Harris, R.; Homeyer, N.; Huang, Y.; Izadi, S.; Kovalenko, A.; Kurtzman, T.; Lee, T. S.; LeGrand, S.; Li, P.; Lin, C.; Liu, J.; Luchko, T.; Luo, R.; Mermelstein, D. J.; Merz, K. M.; Miao, Y.; Monard, G.; Nguyen, C.; Nguyen, H.; Omelyan, I.; Onufriev, A.; Pan, F.; Qi, R.; Roe, D. R.; Roitberg, A.; Sagui, S.; Schott-Verdugo, J.; Shen, C. L.; Simmerling, J.; Smith, R.; Salomon-Ferrer, J.; Swails, J.; Walker, R. C.; Wang, J.; Wei, H.; Wolf, R. M.; Wu, X.; Xiao, L.; York, D. M.; Kollman, P. A. AMBER 2022.

(6) He, X.; Liu, S.; Lee, T. S.; Ji, B.; Man, V. H.; York, D. M.; Wang, J. Fast, Accurate, and Reliable Protocols for Routine Calculations of Protein-Ligand Binding Affinities in Drug Design Projects Using AMBER GPU-TI with ff14SB/GAFF. *ACS Omega* **2020**, *5* (9), 4611–4619. https://doi.org/10.1021/acsomega.9b04233.

(7) Duboué-Dijon, E.; Hénin, J. Building Intuition for Binding Free Energy Calculations: Bound State Definition, Restraints, and Symmetry. *J. Chem. Phys.* **2021**, *154* (20), 204101. https://doi.org/10.1063/5.0046853.

(8) Gallicchio, E.; Levy, R. M. Recent Theoretical and Computational Advances for Modeling Protein–Ligand Binding Affinities. In *Advances in Protein Chemistry and Structural Biology*; Elsevier, 2011; Vol. 85, pp 27–80. https://doi.org/10.1016/B978-0-12-386485-7.00002-8.

(9) Remmert, M.; Biegert, A.; Hauser, A.; Söding, J. HHblits: Lightning-Fast Iterative Protein Sequence Searching by HMM-HMM Alignment. *Nat. Methods.* **2012**, *9* (2), 173–175. https://doi.org/10.1038/nmeth.1818.

(10) Fernandez, C. A.; Smith, C.; Yang, W.; Daté, M.; Bashford, D.; Larsen, E.; Bowman, W. P.; Liu, C.; Ramsey, L. B.; Chang, T.; Turner, V.; Loh, M. L.; Raetz, E. A.; Winick, N. J.; Hunger, S. P.; Carroll, W. L.; Onengut-Gumuscu, S.; Chen, W.-M.; Concannon, P.; Rich, S. S.; Scheet, P.; Jeha, S.; Pui, C.-H.; Evans, W. E.; Devidas, M.; Relling, M. V. HLA-DRB1*07:01 Is Associated with a Higher Risk of Asparaginase Allergies. *Blood* **2014**, *124* (8), 1266–1276. https://doi.org/10.1182/blood-2014-03-563742.
